# Noninvasive proteomic biomarkers for alcohol-related liver disease

**DOI:** 10.1038/s41591-022-01850-y

**Published:** 2022-06-02

**Authors:** Lili Niu, Maja Thiele, Philipp E. Geyer, Ditlev Nytoft Rasmussen, Henry Emanuel Webel, Alberto Santos, Rajat Gupta, Florian Meier, Maximilian Strauss, Maria Kjaergaard, Katrine Lindvig, Suganya Jacobsen, Simon Rasmussen, Torben Hansen, Aleksander Krag, Matthias Mann

**Affiliations:** 1 Novo Nordisk Foundation Center for Protein Research, Faculty of Health and Medical Sciences, University of Copenhagen, Copenhagen, Denmark; 2 Department of Proteomics and Signal Transduction, Max Planck Institute of Biochemistry, Martinsried, Germany; 3 Odense Liver Research Centre, Department of Gastroenterology and Hepatology, Odense University Hospital, Odense, Denmark; 4 Department of Clinical Research, University of Southern Denmark, Odense, Denmark; 5 OmicEra Diagnostics, Planegg, Germany; 6 Functional Proteomics, Jena University Hospital, Jena, Germany; 7 Novo Nordisk Foundation Center for Basic Metabolic Research, Faculty of Health and Medical Sciences, University of Copenhagen, Copenhagen, Denmark; 8 Present Address: Big Data Institute, Nuffield Department of Medicine, University of Oxford, Oxford, UK; 9 Present Address: Pfizer Worldwide Research and Development, San Diego, CA USA

**Keywords:** Proteomics, Mass spectrometry, Biomarkers, Alcoholic liver disease

## Abstract

Alcohol-related liver disease (ALD) is a major cause of liver-related death worldwide, yet understanding of the three key pathological features of the disease—fibrosis, inflammation and steatosis—remains incomplete. Here, we present a paired liver–plasma proteomics approach to infer molecular pathophysiology and to explore the diagnostic and prognostic capability of plasma proteomics in 596 individuals (137 controls and 459 individuals with ALD), 360 of whom had biopsy-based histological assessment. We analyzed all plasma samples and 79 liver biopsies using a mass spectrometry (MS)-based proteomics workflow with short gradient times and an enhanced, data-independent acquisition scheme in only 3 weeks of measurement time. In plasma and liver biopsy tissues, metabolic functions were downregulated whereas fibrosis-associated signaling and immune responses were upregulated. Machine learning models identified proteomics biomarker panels that detected significant fibrosis (receiver operating characteristic–area under the curve (ROC–AUC), 0.92, accuracy, 0.82) and mild inflammation (ROC–AUC, 0.87, accuracy, 0.79) more accurately than existing clinical assays (DeLong’s test, *P* < 0.05). These biomarker panels were found to be accurate in prediction of future liver-related events and all-cause mortality, with a Harrell’s *C*-index of 0.90 and 0.79, respectively. An independent validation cohort reproduced the diagnostic model performance, laying the foundation for routine MS-based liver disease testing.

## Abstract (web summary)

Interogation of mass-spectrometry-based proteomics of liver and plasma from a cohort of patients with alcohol-related liver disease identifies noninvasive biomarkers associated with early stages of disease progression, including significant fibrosis, inflammation and steatosis.

## Main

ALD is a common chronic liver disease that frequently leads to cirrhosis^1^. With rising incidence rates, ALD has become the leading indication of liver transplantation^2^ and is responsible for more than half of all liver-related deaths^3,4^. ALD progresses through a range of histological lesions, starting with alcohol-related fatty liver, to subclinical steatohepatitis featuring hepatic inflammation, which drives progressive fibrosis ultimately leading to cirrhosis. Approximately 75% of patients with ALD are currently diagnosed after decompensated cirrhosis has occurred, making them ineligible for optimal pharmaceutical treatment of alcohol use disorder^5^. Detection of ALD at an early, asymptomatic stage could provide opportunities for slowing or preventing disease progression^6,7^ via intensified alcohol rehabilitation treatment and treatment for metabolic comorbidity, which is known to aggravate hepatic inflammation and steatosis^8^. Early detection of ALD could also have socioeconomic benefits, as seen with very cost-effective early detection of liver fibrosis in primary care when using the enhanced liver fibrosis (ELF) blood test combined with imaging-based tests^9^.

However, the slow and asymptomatic nature of disease progression renders diagnosis at an early stage challenging. Accurate diagnosis of liver disease requires biopsy, a procedure that causes major complications in 1% of cases^10^, and existing noninvasive biomarkers have limited accuracy in the early disease stages, thus severely reducing opportunities for timely disease detection and intervention^11^. Therefore, there is a pressing need for minimally invasive diagnostic strategies to screen patients in at-risk populations such as individuals with a history of alcohol misuse, obesity, inactivity and diabetes. Prognostic markers could help patients with ALD through better disease management to avoid decompensated cirrhosis. Furthermore, the molecular pathophysiology of ALD is incompletely understood and there are currently no liver-specific interventions against the condition. Characterization of liver and plasma proteome dynamics across the full spectrum of disease could provide new biological insights into disease mechanisms and provide insights into new therapeutic options.

Biomarker discovery efforts have typically focused on individual biomolecules^12,13^ and have had a low acceptance rate in the clinic. Systems-wide studies would be attractive, especially if they could connect circulating levels and dysregulation in the diseased organ^14,15^. Such global data can be used to build machine-learning-based classification models^16,17^.

Recent advances in MS-based proteomics have greatly extended its reach in biomedical and clinical research^18–20^. It enables specificity in the identification and quantification of hundreds to thousands of proteins present in biological or clinical samples, making it suitable in principle for the study of disease mechanisms and identification of biomarkers. In the context of complex diseases, MS-based proteomics could shift the focus to biomarker panels rather than individual proteins. However, to be effective as a clinical biomarker discovery platform, MS-based proteomics has to be performed in a robust and accurate manner and applied to large patient cohorts. To this end, our group has developed a plasma proteome-profiling workflow with which we have already identified circulating proteins associated with nonalcoholic fatty liver disease (NAFLD)^21,22^.

Here, we used MS-based proteomics to analyze paired liver tissue and plasma samples, and clinical outcomes, from a large cohort of patients with a history of alcohol misuse and with asymptomatic ALD representing early stages on the disease spectrum, and from healthy controls with age and gender matched to the ALD cohort, totaling 596 participants. We then validated the findings in an independent ALD cohort. Our goal was to systematically characterize proteome changes in liver and plasma in a pathological feature- and disease-stage-dependent manner by performing separate and integrative analysis of the two tissue types. In addition, we aimed to explore the potential of plasma proteomics as a clinical diagnostic and prognostic tool for liver disease by assessing its predictive capability in the context of existing best-in-class clinical tests^12,23,24^. We hypothesized that MS-based plasma proteomics could identify biomarker panels of diagnostic and prognostic value in the clinic, and that analysis of matched liver tissue and plasma proteomes could reveal insights about the pathophysiology of ALD and determine the tissue origin of potential circulating biomarkers.

We observed that both liver and plasma proteomes undergo extensive remodeling during ALD, with fibrosis having the largest effect followed by hepatic inflammation and steatosis. By the application of machine learning to plasma proteomics data, we defined biomarker panels for the simultaneous detection of ‘significant’ fibrosis (fibrosis stage ≥F2), mild inflammatory activity and any steatosis. We benchmarked these biomarker panel-based models against 15 existing tests in the discovery cohort and 11 tests in the validation cohort that demonstrated superior or comparable performance to in-class comparators for significant fibrosis and mild inflammatory activity (judged by F1 score, balanced accuracy and/or ROC–AUC). Extraction of patient follow-up data from electronic health records further demonstrated high prognostic performance for liver-related events and all-cause mortality. We provide an open-source interactive data visualization tool for data exploration (Supplementary Fig. 1).

## Results

### Patients, data collection and study design

The study population consisted of participants from three cohorts: (1) gut-and-liver axis–alcohol-related liver disease (GALA–ALD), termed the derivation cohort (*n* = 459, prospective cohort with follow-up data available); (2) gut-and-liver axis–healthy participants (GALA–HP), a cross-sectional cohort of healthy controls matched to GALA–ALD from the GALAXY Horizon2020 consortium (livergalaxy.eu) (*n* = 137); and (3) the independent ALD validation cohort (*n* = 63, a cross-sectional cohort from a screening study for ALD (clinicaltrials.gov ID NCT03308916; https://open.rsyd.dk/OpenProjects/openProject.jsp?openNo=475&lang=da), with patients receiving a liver biopsy in the case of elevated liver stiffness as evidence of liver fibrosis) (Table 1).

**Table 1:** Baseline participant characteristics

Variable	GALA–ALD (*n* = 459)	GALA–HP (*n* = 137)	ALD validation cohort (*n* = 63)
Age	57 ± (13)	53 ± (13)	58 ± (14)
BMI (kg m^–2^)	27.4 ± (6.7)	26.1 ± (4.7)	30.2 ± (8.1)
Male gender	349 (76%)	86 (63%)	54 (86%)
Female gender	110 (24%)	51 (37%)	9 (14%)
ALT (U l^–1^)	31 ± (26)	24 ± (11)	41 ± (35.5)
AST (U l^–1^)	34 ± (26)	25 ± (7)	34 ± (31.5)
Alkaline phosphatase (U l^–1^)	80 ± (44)	64 ± (21)	83 ± (31.5)
GGT (U l^–1^)	72 ± (156)	22 ± (12)	97 ± (222.3)
Albumin (g l^–1^)	42 ± (5)	44 ± (3)	45 ± (4)
Bilirubin (µmol l^–1^)	10 ± (7)	11 ± (6)	8.5 ± (6)
Platelet count (10^9^ l^–1^^)^	232 ± (100)	236 ± (61)	212.5 ± (73.8)
MELD score	6 ± (2)	6 ± (1)	7 ± (2)
HbA1C (mmol mol^–1^)	36 ± (6)	35 ± (5)	37 ± (9)
Total cholesterol (mmol l^–1^)	5 ± (1.6)	5.2 ± (1.1)	4.8 ± (1.9)
Drinking history (years of excessive drinking)	16 ± (18)	NA	15 ± (21)
ELF	9.3 ± (2)	NA	10 ± (1.4)
FIB-4 index	1.5 ± (1.5)	NA	1.6 ± (1.4)
TE (kPa)	6.5 ± (6.8)	4.3 ± (1.7)	9.2 ± (5.4)
2D-SWE (kPa)	8.3 ± (8.4)	5.5 ± (1.3)	9 ± (7.4)
Abstaining from alcohol at time of inclusion	192 (42%)	13 (9%)	21 (33%)
Fibrosis stage 0/1/2/3/4	36/124/106/27/67	NA	4/15/21/13/9
Steatosis 0/1/2/3	156/85/72/39	NA	19/20/12/8
Ballooning 0/1/2	178/108/66	NA	37/16/6
Lobular inflammation 0/1/2/3	80/160/84/28	NA	10/30/18/1
Ballooning + lobular inflammation 0/1/2/3/4/5	72/91/82/53/31/23	NA	10/21/13/10/5

All summary data are medians ± interquartile range (IQR) or sums with proportions. Histological staging was performed according to the NAFLD activity score (NAS); 98 patients in the GALA–ALD cohort did not have a biopsy due to low liver stiffness (FibroScan <6 kPa). GGT, gamma-glutamyl transferase. NA, not applicable.

**Fig. 1:**
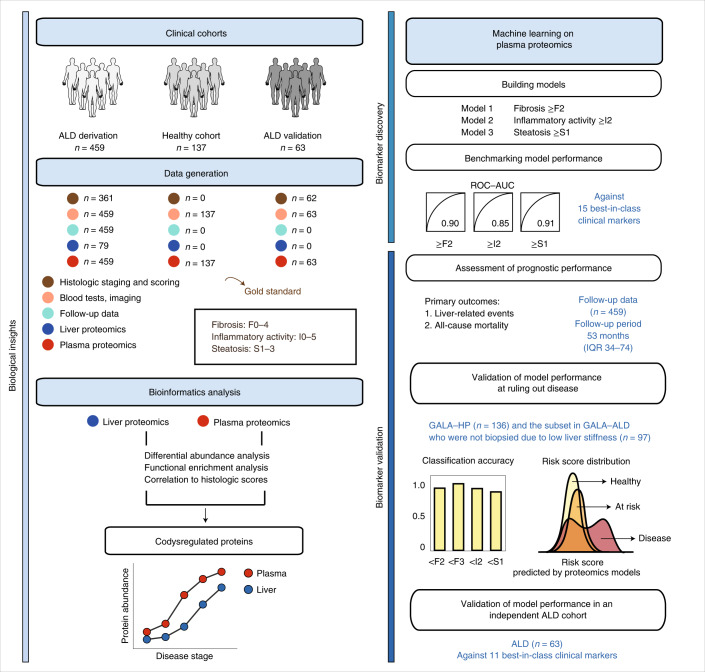
A framework for biomarker discovery in liver disease. High-throughput, MS-based proteomics technology used to profile paired liver and plasma samples from 459 patients with ALD and 137 matched healthy controls. Proteome dysregulation in liver and plasma were integrated to capture disease-stage-relevant protein signatures in the bloodstream that were concordant with the liver. Last, a machine learning model was built to identify early stages of liver fibrosis, inflammatory activity and steatosis. We used the diagnostic models to assess their prognostic capabilities. We also validated model performance to rule out disease in low-incidence populations. In addition, the diagnostic capability of identified protein marker panels was evaluated in an independent cohort of 63 patients with ALD.

The GALA–ALD cohort is a diagnostic-test cohort of consecutively recruited patients with a history of harmful drinking. They represent asymptomatic ALD within the full spectrum of early stages of the disease, because any individuals with known chronic liver disease and/or clear signs of decompensated, late-stage disease were excluded. Investigations on a subset of the cohort are described in detail elsewhere^12,23,24^. The same pathologist scored all liver biopsies, blinded to clinical patient information, according to the NAFLD activity score–clinical reasearch network (NAS–CRN)^25^ for fibrosis (F0–4), lobular inflammation (0–3), ballooning (0–2) and steatosis (0–3). We defined inflammatory activity as the sum of lobular inflammation and ballooning. We used the derivation cohort for bioinformatics analysis, machine-learning-based biomarker panel derivation for significant fibrosis (≥F2), mild inflammatory activity (≥I2) and any steatosis (≥S1), and assessment of the prognostic ability of the derived marker panels. The GALA–HP and validation cohorts were used for validation of the ability of the proteomics biomarker panels to rule out and rule in disease (Fig. 1). Detailed participant recruitment inclusion and exclusion criteria can be found for all cohorts in Methods.

**Extended Data Fig. 1:**
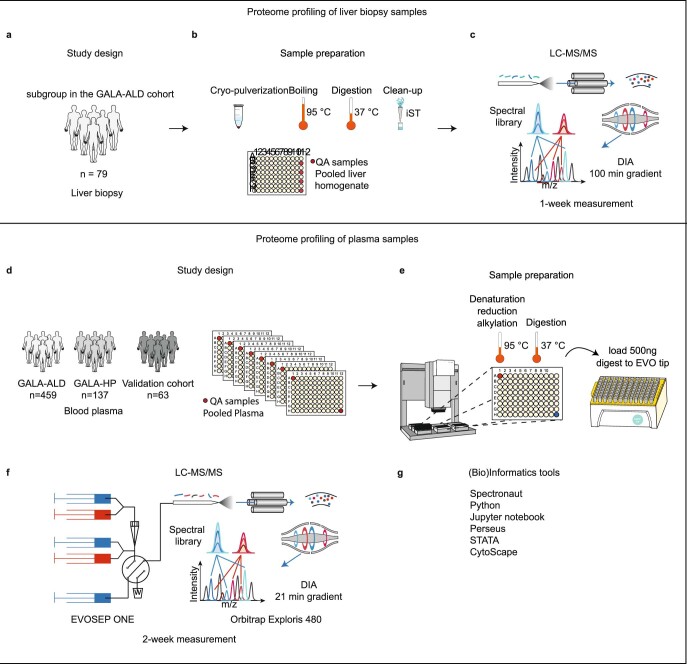
Proteomics workflow. **a-c**. Proteome profiling workflow for liver biopsy samples. **d-f**. Proteome profiling workflow for plasma samples. **g**. Computational and bioinformatics tools used for processing and analyzing proteomics data.

**Extended Data Fig. 2:**
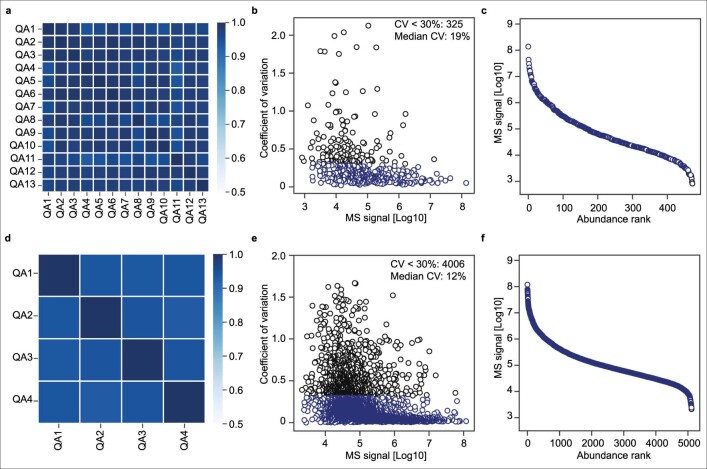
Proteomics data quality. **a,d**. Pair-wise Pearson correlation between proteomes of the workflow replicates in the plasma (a) and liver (d) proteomics experiments. **b, e**. The coefficients of variation (CV) of each protein assessed by quality assessment samples are plotted against their median intensity, with (b) showing the plasma- and (e) showing the liver proteomics experiment. **c, f**. Protein intensity as a function of abundance rank in the plasma- and liver proteomes (c and f, respectively). Source data

**Extended Data Fig. 3:**
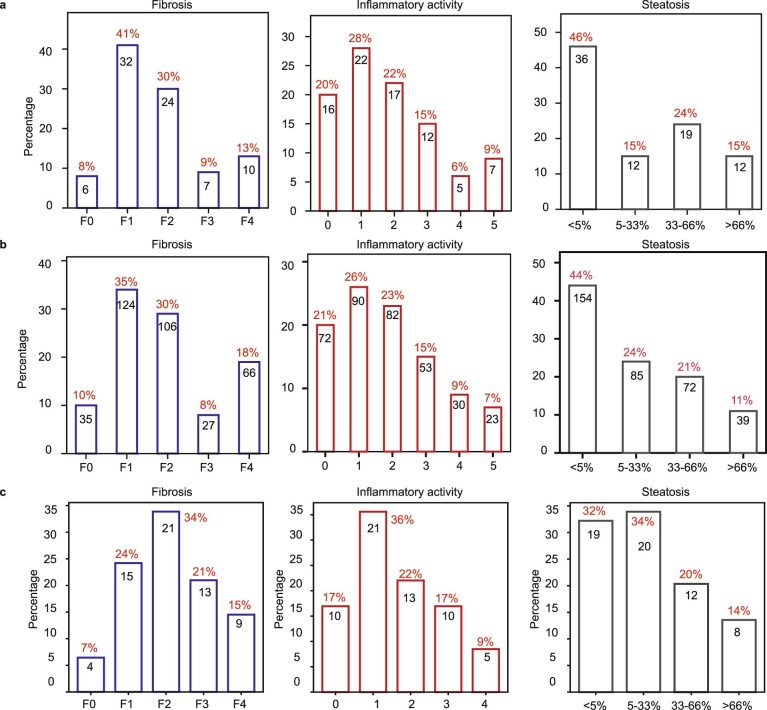
Histologic score distributions. **a**. Stage distribution of fibrosis (based on Kleiner score), inflammatory activity (summed lobular inflammation and ballooning scores) and steatosis (percentage of hepatic fat content) in patients whose liver biopsy proteomes were analyzed (n = 79). **b**. Stage distribution of fibrosis, inflammatory activity, and steatosis in patients whose plasma proteome were analyzed and passed quality control (n = 358). **c**. Stage distribution of fibrosis, inflammatory activity, and steatosis in the independent validation cohort of ALD (n = 63). Note that the number of patients in each sub-group may not add up to the total number due to missing histological scores.

We acquired plasma proteome profiles of all participants (*n* = 659) using a data-independent acquisition (DIA) strategy^26^ and a single-run workflow on an Evosep One liquid chromatography system^27^ (Evosep Biosystems) coupled online to an Orbitrap Exploris 480 mass spectrometer (Thermo Fisher Scientific). In addition, we analyzed 79 liver biopsy proteomes in the derivation cohort with a DIA method in 100-min gradients, quantifying 5,515 proteins in total. Both plasma and liver proteomics data were acquired using optimized methods enabled by MaxQuant.Live^28^ with a signal processing algorithm termed PhiSDM^29^ for rapid liquid chromatography–tandem MS (LC–MS/MS) cycle times. An overview of proteomics workflow and dataset quality can be found in Extended Data Figs. 1 and 2. Stage distribution of fibrosis, inflammatory activity and steatosis in the liver and the plasma proteomics dataset in the derivation cohort, as well as that in the plasma proteomics dataset in the validation cohort, can be found in Extended Data Fig. 3a–c, respectively.

We benchmarked the model performance against 15 of the most widely validated, commercially available, European Medicines Agency/Food & Drug administration (FDA)-approved and widely used imaging and serum tests for liver fibrosis, inflammation and steatosis in the derivation cohort: transient elastography^24,30^ (TE; FibroScan, Echosens), two-dimensional shear-wave elastography^24^ (2D-SWE), the ELF blood test^12,31,32^, P3NP^31,33,34^, FibroTest^12,35^, the Fibrosis-4 (FIB-4) index^36^, the Forns index^37^ and the AST/platelet ratio index (APRI)^38^ for fibrosis; cytokeratin-18-based markers M30 and M65 and their ratio M30/M65 (ref. ^39^), together with alanine aminotransferase (ALT)^40^, aspartate aminotransferase (AST)^40^ and AST/ALT ratio (AAR)^41^ for inflammation; controlled attenuation parameter (CAP) for steatosis; and 11 of the above tests in the validation cohort.

### Impact of hepatic lesions on liver and plasma proteomes

**Fig. 2:**
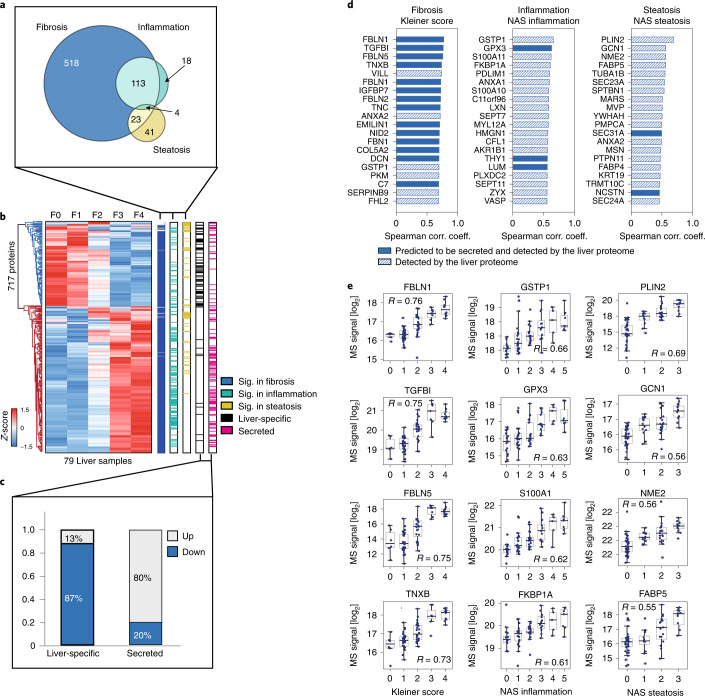
Liver proteome remodeling due to hepatic lesions. **a**, Proteins in liver tissue that were significantly differentially abundant across stages of fibrosis, inflammatory activity and steatosis (FDR-corrected *P* < 0.05). *n* = 6/32/24/7/10 biologically independent samples for Kleiner score 0/1/2/3/4; *n* = 16/22/17/12/5/7 biological independent samples for NAS inflammation score 0/1/2/3/4/5; and *n* = 36/12/19/12 for NAS steatosis score 0/1/2/3. **b**, Hierarchical clustering of all significantly (sig.) dysregulated proteins in the liver proteome. Row clustering was based on median log_2_ intensity after *z*-score normalization across fibrosis stages F0–4. **c**, Fraction (%) of up- and downregulated liver-specific and secreted proteins. **d**, Top 20 proteins that correlate with Kleiner, NAS inflammation and NAS steatosis scores, respectively. Number of independent biological replicates for each disease group is the same as in **a**. **e**, Distribution of log_2_ intensity values of top four proteins correlating to each histologic score. The gray line in the middle of the box is the median, the top and bottom of the box represent the upper and lower quartile values of the data and the whiskers represent the upper and lower limits for consideration of outliers (Q3 + 1.5 × IQR, Q1 – 1.5 × IQR). IQR represents IQR (Q3 – Q1). Source data

Differential abundance analysis in the liver proteome (Methods) revealed 658 proteins significantly dysregulated (false discovery rate (FDR)-adjusted *P* < 0.05) across stages of fibrosis, 135 of inflammatory activity and 68 of steatosis, totaling 717 unique proteins accounting for 13% of the total quantified liver proteome (Fig. 2a and Supplementary Table 1). Fibrosis was associated with the largest effect on the liver proteome, followed by inflammation, and 84% of all proteins dysregulated in inflammation were also found to be dysregulated in fibrosis. The majority of dysregulated proteins were upregulated as fibrosis stage increased (63% (454 proteins) versus 37% (263 proteins) downregulated; Fig. 2b). When grouping dysregulated proteins according to annotations of the Human Protein Atlas^42^, 87% of the proteins annotated as ‘liver specific’ (Methods) were downregulated in liver tissue as fibrosis stage increased; in contrast, 80% of the proteins annotated as ‘secreted’ were upregulated (Fig. 2c). This is similar to what we previously reported in a study of NAFLD and cirrhosis^21^.

Approximately 25% of the upregulated proteins belong to the immune system (Supplementary Table 2). Signal transduction pathways were the second most highly over-represented category as fibrosis progressed (22% of total upregulated proteins) and receptor tyrosine kinase signaling was upregulated, in agreement with existing literature that both TGF-β and platelet-derived growth factor play a role in fibrogenesis^43^. Specifically, MAPK1, MAPK3, ROCK2 and AKT2, phosphatases PTPN6 and PTPN11, transcription factor STAT1 and small GTPases RAC1 and RAP1B were upregulated between fibrosis stages F0 and F4 (Supplementary Table 1). Signaling by Rho GTPase (40 proteins) and GPCR (22 proteins) were also significantly upregulated. Additionally, proteins in extracellular matrix (ECM) organization were significantly upregulated (44 proteins, including collagen types I, III, IV, V, VI, XII and XIV, fibronectin, laminin, lumican, perlecan, fibulins FBLN1, FBLN2 and FBLN5 and latent-transforming growth factor beta-binding proteins LTBP1 and LTBP4). Proteins observed to be downregulated were largely metabolism related (158 proteins, 60%; Supplementary Table 3).

Correlation analysis (Methods) resulted in 1,235 proteins significantly correlated with stages of fibrosis, 873 with inflammatory activity and 175 with steatosis score (Supplementary Tables 4–6). Among the top 20 proteins most correlated with stages of fibrosis were prominent liver-secreted proteins (Fig. 2d) with roles in cell–ECM interactions (TGFBI and EMILIN1 (ref. ^44^)), as well as those previously associated with hepatic fibrosis (IGFBP7 (ref. ^45^) and TNXB^46^). Approximately 50% of the top 20 inflammation-associated proteins are cytoskeletal proteins, potentially indicating cellular structural changes in the inflamed liver (Fig. 2d), while the lipid droplet protein PLIN2 and fatty acid-binding protein 4 (FABP4), an adipokine previously identified as a predictive marker for progression from simple steatosis to nonalcoholic steatohepatitis in patients with NAFLD^47^, had the first and 16th highest correlation coefficients, respectively, to hepatic steatosis. The top four proteins correlated with each histological score are shown in Fig. 2e.

**Extended Data Fig. 4:**
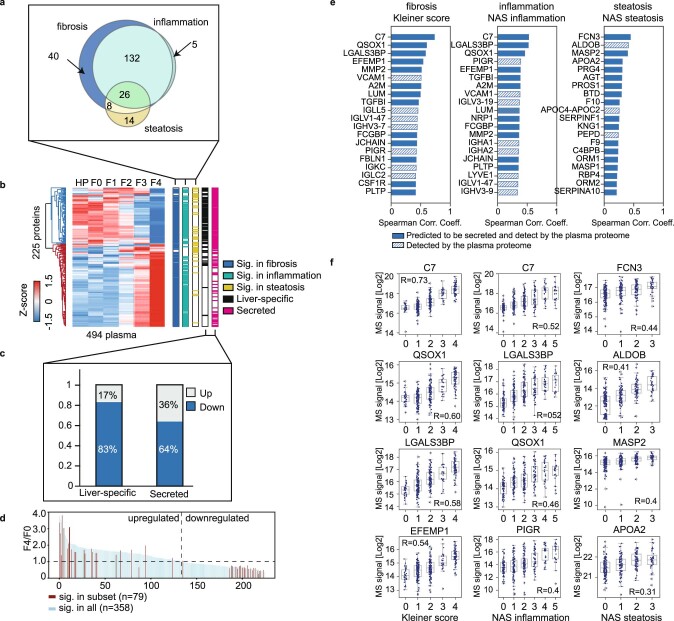
Remodeled plasma proteome due to hepatic lesions. **a**. Proteins in plasma significantly differentially abundant across stages/scores of fibrosis, inflammatory activity and steatosis in the disease cohort with biopsy-verified histologic scores (FDR-corrected p-value < 0.05). n = 35/124/106/27/66 biologically independent samples for Kleiner score 0/1/2/3/4; n = 72/90/82/53/30/23 biological independent samples for NAS inflammation score 0/1/2/3/4/5; and n = 154/85/72/39 for NAS steatosis score 0/1/2/3. **b**. Hierarchical clustering of significantly dysregulated plasma proteins. Row clustering was based on median log2-intensity after Z-score normalization across fibrosis stages HP-F0-F4. **c**. Ratios of up- and downregulated proteins of ‘liver-specific’ and ‘secreted’ proteins. **d**. Fold change (fibrosis stage F4/F0) of significant proteins when using the whole dataset (n = 358) and the subset with paired liver proteomes available (n = 79) **e**. Top 20 plasma proteins that correlate with the Kleiner score, inflammatory activity, and steatosis stages. **f**. Distribution of log2-intensity values of top four correlating proteins in plasma for each histologic score. Number of independent biological replicates is the same as Panel (a). The gray line in the middle of the box is the median, the top and the bottom of the box represent the upper and lower quartile values of the data and the whiskers represent the upper and lower limits for considering outliers (Q3 + 1.5*IQR, Q1‐1.5*IQR). IQR is the interquartile range (Q3–Q1). Source data

In total, 225 proteins were significantly differentially abundant across histologic stages of fibrosis (206 proteins), inflammatory activity (163 proteins) and steatosis (48 proteins), underlying remodeling of the plasma proteome as a function of liver pathology (Extended Data Fig. 4a and Supplementary Table 7). Among these, 134 proteins were up- and 91 downregulated as fibrosis stage increased (Extended Data Fig. 4b). Of those dysregulated proteins annotated as liver specific or secreted, 83 and 64%, respectively, were found to be downregulated (Extended Data Fig. 4c). The downregulated cluster includes proteins involved in the complement system (complement 4A, 6, 8B, 8G and 9), coagulation cascade (coagulation factors F2, F5, F7, F9, F10, F11, F13A1, PROC, PROS1, PROZ and SERPINC1), apolipoproteins (APOA1, APOA2, APOB, APOC1, APOC3, APOC4, APOF, APOH, APOL1 and APOM) and carrier proteins (ALB, TTR, GC, RBP4 and HPX) (Supplementary Table 7). To demonstrate the importance of sufficient statistical power in human proteomics studies, we performed analysis of covariance (ANCOVA) on the subset of 79 plasma samples whose paired liver proteomes were also measured. This resulted in only 45 significantly differentially abundant proteins (Supplementary Table 8), 84% of which were among the top 100 most dysregulated proteins (F4/F0) when using the complete dataset (*n* = 358), indicating that proteins with a larger effect size are more robust to reduced sample size (Extended Data Fig. 4d).

Circulating levels of 106 proteins significantly correlated to fibrosis stages, 55 to inflammatory activity and five to steatosis (Supplementary Tables 9–11). Complement component C7 (Spearman *r* = 0.73), QSOX1 (*r* = 0.6) and LGALS3BP (*r* = 0.58) showed the highest correlation to fibrosis stages (also, the top three correlated to inflammation) (Extended Data Fig, 4e,f), representing promising fibrosis and inflammation marker candidates due to their roles in ECM remodeling, immune response and disease stage-dependent increase in the circulation. Fructose biphosphate aldolase B (ALDOB), a key enzyme in aldolase metabolism and a potential biomarker for NAFLD^21^, had the second highest correlation to steatosis (*r* = 0.41) (Extended Data Fig. 4f).

### Integration of liver and plasma proteomics

**Fig. 3:**
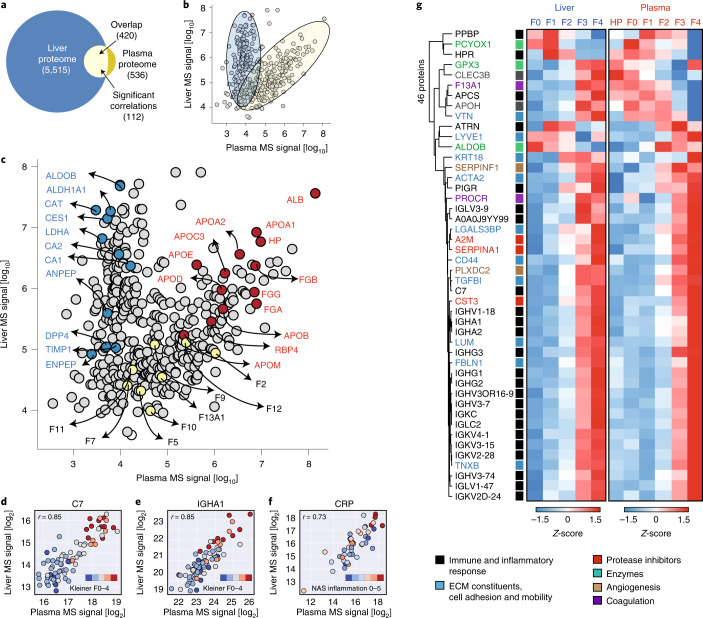
Liver–plasma proteome integration. **a**, Overlapping proteins between liver and plasma proteomes. The number of proteins significantly correlated between liver and plasma across patients is denoted (FDR-corrected *P* < 0.05 and minimum absolute Pearson *r* = 0.3). **b**, Liver–plasma proteome abundance map showing median protein intensity (assessed by MS intensity) in the liver as a function of that in the plasma. **c**, As in **b** but highlighting enzymes, clotting factors and functional plasma proteins known to be related to liver. **d**–**f**, Represented proteins (C7 (**d**), IGHA1 (**e**) and CRP (**f**)) were significantly correlated in paired liver and plasma samples, with Pearson *r* indicated. **g**, Top: proteins codysregulated in liver and plasma during disease progression; the dendrogram shows significantly codysregulated proteins across histologic stages of liver fibrosis, inflammation and steatosis. Bottom: functional categorization of proteins. Heat maps display *z*-scored median intensities across fibrosis stages within liver (left) and plasma (right). Source data

**Fig. 4:**
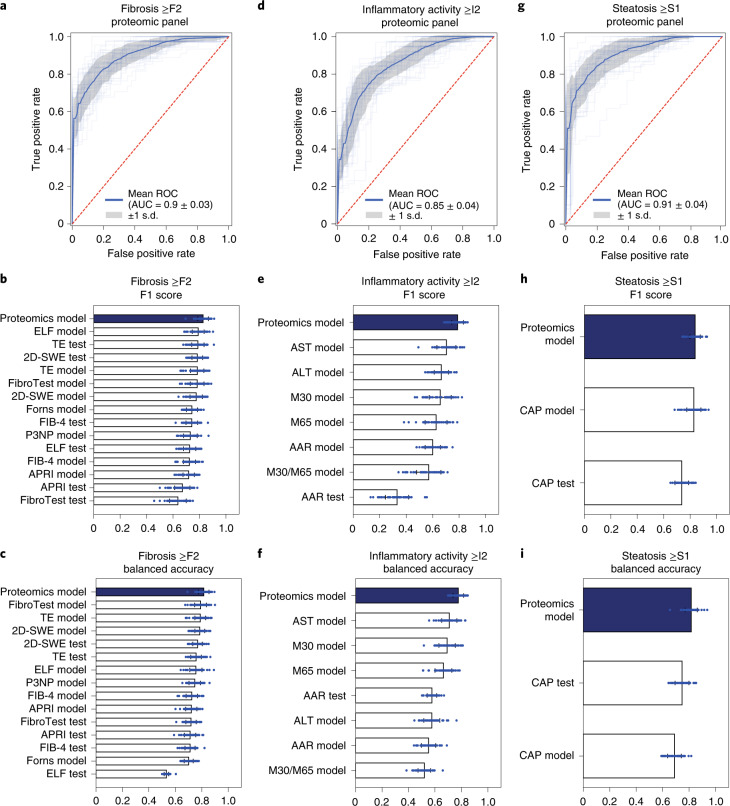
Prediction models based on plasma proteome for biopsy-verified fibrosis, inflammation and steatosis. **a–i**, Prediction models. **a**,**d**,**g**. ROC–AUC statistics in fivefold cross-validation repeated ten times in a protein panel-based logistic regression model for detection of significant liver fibrosis (F2–4) (**a**), mild inflammatory activity (NAS I2–5) (**d**) and any steatosis (NAS S1–3) (**g**). **b**,**c**,**e**,**f**,**h**,**i**, F1 score (**b**,**e**,**h**) and balanced accuracy (**c**,**f**,**i**) in cross-validation of the above-mentioned logistic regression models in comparison with best-in-class existing markers for fibrosis (**b**,**c**), inflammation (**e**,**f**) and steatosis (**h**,**i**). Performance of existing markers was calculated based on both their established clinical cutoffs if applicable (indicated by ‘test’) and machine learning cutoffs (indicated by ‘model’). Error bars represent s.d. **b**,**c**,**e**,**f**,**h**,**i**, *n* = 50 independent experiments in the fivefold 10× cross-validation procedure; data presented as mean ± s.d. Source data

In total, 420 proteins were commonly quantified in the liver and plasma (Fig. 3a). Their levels in a 2-dimensional space exhibited a bimodal pattern. For one group of proteins, relative abundance rank in liver and plasma were largely correlated (‘diagonal cluster’) whereas for the other group there was no correlation (‘vertical cluster’, where each dot represents a protein; Fig. 3b). For example, liver enzymes ALDOB and DPP4 exhibited about 1,000-fold difference in abundance in the liver whereas their levels in plasma were low and very similar (Fig. 3c). In general, intracellular enzymes were part of the vertical cluster, including ALDOB, CES1, ALDH1A1 and LDHA, which were detected in the plasma at very low levels—10,000-fold less than albumin—presumably reflecting tissue leakage. The diagonal cluster represents plasma proteins serving many different functions, including highly abundant proteins such as albumin, apolipoproteins A1, A2 and C3, hepatoglobin, SERPINA1 and fibrinogen subunits, as well as less abundant examples such as coagulation factors F2, F9, F10, F11, F12 and F13A1 (Fig. 4c). Among these commonly detected proteins, 112 had significant correlations between paired liver and plasma samples (Pearson’s *r* up to 0.85 for C7 and IgA1; Fig. 3d,e and Supplementary Table 12). C-reactive protein (CRP) levels in liver tissue and circulation also correlated highly (*r* = 0.73), which is of interest given the widespread use of this protein as a systemic risk marker for cardiovascular disease (Fig. 3f).

In total, 46 proteins were commonly differentially abundant in ANCOVA of the liver and plasma proteome (for simplicity, we hereinafter refer them as codysregulated proteins) (Fig. 3g and Supplementary Table 13). These represent immune and inflammatory responses, cell adhesion, ECM organization, protease inhibitors and intracellular enzymes (Fig. 3g). They are codysregulated probably due to immune cell infiltration and increased overall systemic inflammation, ECM remodeling and scar tissue formation in liver fibrosis, and tissue leakage. Most codysregulated proteins increased in abundance from fibrosis stage F0 to F4 in both liver and plasma, including PIGR, LGALS3BP, TGFBI and C7, all promising markers in liver disease that have previously been reported^21^. A few exceptions, such as tetranectin (CLEC3B), vitronectin (VTN), F13A1 and APCS, exhibited the opposite patterns in liver and plasma as fibrosis increased. Apart from these monotonic patterns, ALDOB increased from F0 to F2 followed by a decrease in advanced fibrosis in the plasma, but continuously decreased from F0 to F4 in the liver, consistent with our previous observation in NAFLD and cirrhosis^21^. Decrease of hepatic ALDOB may be the result of reduced functional hepatocyte cell mass, while plasma levels seem to be a combined effect of tissue leakage (similar to liver damage markers ALT and AST) and impaired liver synthesis.

### Biomarker panels for early-stage pathology of ALD

**Extended Data Fig. 5:**
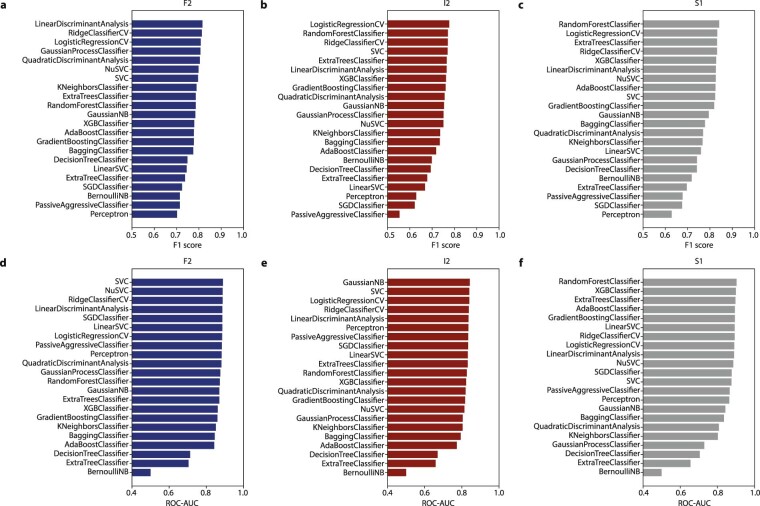
Model performance among state-of-the-art classifiers. **a-c**. The F1 score of all classifiers for predicting significant fibrosis (F2, a), mild inflammatory activity (I2, b), and any steatosis (S1, c). Classifiers were ranked in decreasing order of F1 score. **d-f**. The area under the receiver operating characteristics curve (ROC-AUC) of all classifiers for predicting F2 (d), I2 (e), and S1 (f). Classifiers were ranked in decreasing order of ROC-AUC.

**Extended Data Fig. 6:**
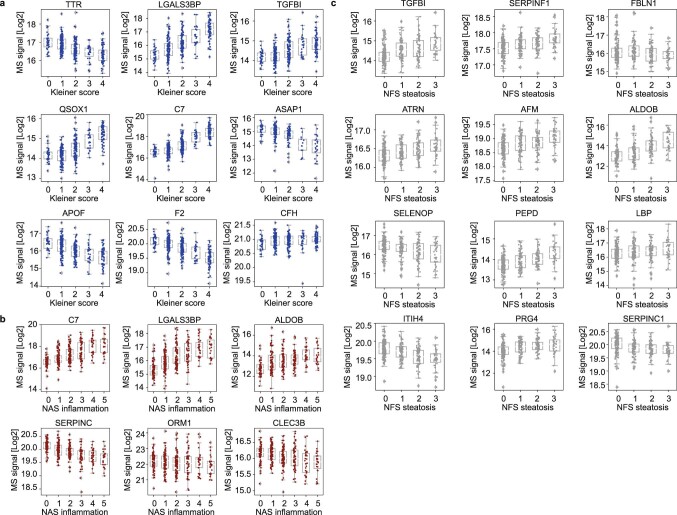
Abundance distribution of proteins in the selected marker panels. **a-c**. Proteins comprising the marker panels for identifying significant fibrosis (a), mild inflammatory activity (b) and any steatosis (c), and their abundance distribution (log2-transformed) as a function of corresponding histologic stages. The gray line in the middle of the box is the median, the top and the bottom of the box represent the upper and lower quartile values of the data and the whiskers represent the upper and lower limits for considering outliers (Q3 + 1.5*IQR, Q1‐1.5*IQR). IQR is the interquartile range (Q3–Q1). For Panel (a), n = 35/124/106/27/66 biologically independent samples for Kleiner score 0/1/2/3/4 except for protein ASAP1 which has n = 30/94/62/13/24; For panel (b), n = 72/90/82/53/30/23 biological independent samples for NAS inflammation score 0/1/2/3/4/5 except for protein ALDOB which has n = 49/72/65/50/29/22. For panel (c), n = 154/85/72/39 for NAS steatosis score 0/1/2/3 except for protein ALDOB which has n = 97/80/71/39. Source data

We first compared 22 state-of-the-art machine learning classifiers and determined a logistic regression model as the final classifier for its overall superior performance, simplicity and interpretability (Methods and Extended Data Fig. 5). Using the ‘minimum redundancy–maximum relevance’ (mRMR)^48^ feature selection algorithm, we selected a nine-protein marker panel for identification of significant fibrosis (≥F2: 200 controls, 160 cases), six for mild inflammatory activity (≥I2: 153 controls, 189 cases) and 12 for any liver steatosis (≥S1: 156 controls, 196 cases). These proteins either increased or decreased following disease progression (Extended Data Fig. 6). Among the 22 unique proteins comprising the three marker panels, eight were codysregulated in both plasma and liver including C7, LGALS3BP, TGFBI, ALDOB, CLEC3B, FBLN1, ATRN and SERPINF1 (Supplementary Table 14). The proportion of codysregulated proteins was highest for the inflammation panel, reaching 67% (33 and 42% for the fibrosis and steatosis panels, respectively). The mRMR algorithm also detected ALDOB, AFM and LGALS3BP, three of the six proteins we previously identified as candidate markers for NAFLD^21^. Logistic regression models based on the selected marker panels had a mean cross-validated ROC–AUC of 0.90 (95% confidence interval (CI) 0.899–0.905), 0.85 (95% CI 0.851–0.858) and 0.91 (95% CI 0.907–0.915) for prediction of significant fibrosis, mild inflammatory activity and any steatosis, respectively (Fig. 4a,d,g and Supplementary Table 15). When compared with the 15 state-of-the-art clinical tests using widely agreed, established cutoffs or logistic regression-determined cutoffs (Supplementary Table 16), all proteomics models had the highest F1 scores and balanced accuracies (Fig. 4b,c,e,f,h,i). A final prediction model based on a new random split resulted in ROC–AUC values of 0.92, 0.87 and 0.85 for identification of the three above-mentioned endpoints (hereinafter referred as the F2, I2 and S1 proteomics models, respectively) (Supplementary Table 17). The F2 proteomics model significantly outperformed logistic regression models using either the Forns index, APRI or the FIB-4 Index (DeLong’s test, *P* < 0.05) and was equally good as the others, including the best-in-class reference, transient elastography (Supplementary Table 18). The I2 proteomics model significantly outperformed logistic regression models using ALT, AAR and the commercial marker M30/M65 ratio, and the S1 proteomics model was equally as good as CAP. Although we focused on early-stage fibrosis, we also built a model for identification of advanced fibrosis (≥F3). It had an ROC–AUC of 0.974 (Supplementary Table 17), which significantly outperformed five clinical tests (Supplementary Table 18). These final proteomics models were used for subsequent validation and assessment of their prognostic capability.

### Validation of model performance in an independent cohort

**Fig. 5:**
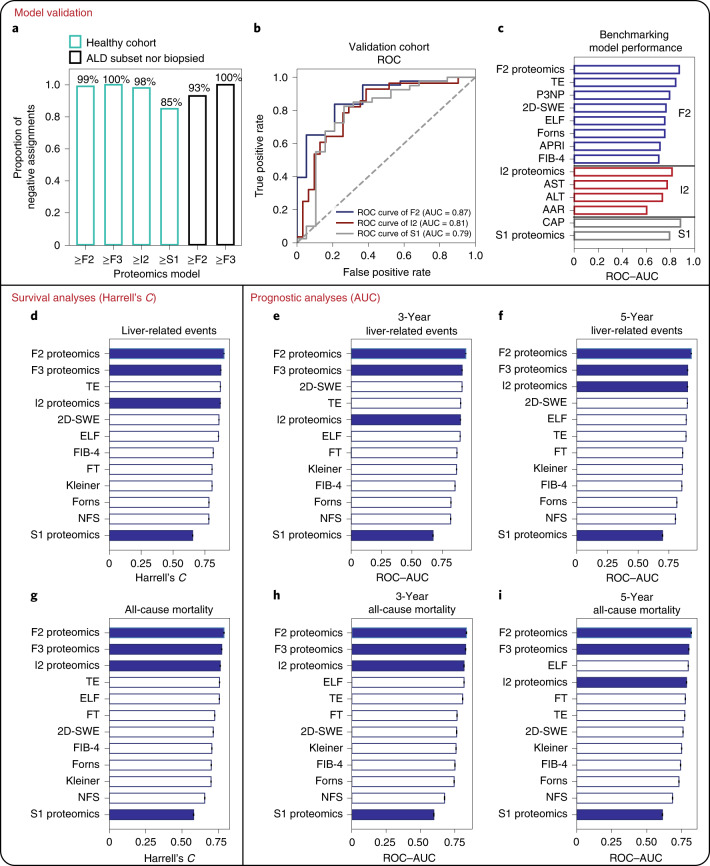
Validation of model performance and assessment of prognostic capability. **a**, Percentage accuracy of proteomics models in excluding significant fibrosis, advanced fibrosis, mild inflammatory activity and steatosis in the healthy cohort (GALA–HP; *n* = 136), and in excluding significant fibrosis and advanced fibrosis in the subset of the GALA–ALD cohort (*n* = 97) who were not biopsied due to low liver stiffness as measured by FibroScan (<6.0 kPa). **b**, ROC curve and the corresponding AUC of F2, I2 and S1 proteomics models for the independent validation cohort (*n* = 63). **c**, ROC–AUC of F2, I2 and S1 proteomics models for the validation cohort in comparison with available best-in-class clinical tests. **d**–**i**, Survival and prognostic analyses of proteomics models. Survival analyses and clinical tests in prediction of future liver-related events (**d**) and all-cause mortality (**g**) during the entire follow-up period, ranked by Harrell’s *C*-index in descending order. **e**,**f**, Prognostic analyses of proteomics models and clinical tests in prediction of 3-year (**e**) and 5-year (**f**) liver-related events, ranked by ROC–AUC in descending order. **h,i**, Prognostic analyses of proteomics models and clinical tests in prediction of 3-year all-cause mortality (**h**) and 5-year all-cause mortality (**i**), ranked by ROC–AUC in descending order. NFS, NAFLD fibrosis score. Source data

The proteomics models correctly excluded significant fibrosis, advanced fibrosis and mild inflammation in the cohort of healthy, matched controls (*n* = 136), with an accuracy of 99, 100 and 98%, respectively (Fig. 5a and Supplementary Table 19). Although around 15% of healthy individuals were classified as steatosis positive, since they were matched for body mass index (BMI) with the GALA–ALD cohort and simple steatosis was not an exclusion criterion, clinical misassignment is possible. Supporting these steatosis-positive assignments by the S1 proteomics model, 65% had a CAP value >290, the threshold for ruling in fatty liver. The high consensus between the S1 proteomics model and CAP, an independent method for detection of liver steatosis, implies a potentially even higher accuracy of the S1 proteomics model in excluding steatosis. Clinically, it is relevant to exclude liver damage in at-risk populations such as alcohol misuse. For the subset of patients with a history of alcohol misuse and who were not biopsied due to low liver stiffness (*n* = 97), the proteomics models correctly excluded significant and advanced fibrosis in 93 and 100% of cases, respectively (Fig. 5a and Supplementary Table 19). However, liver stiffness based on transient elastography is only moderately accurate for exclusion of significant fibrosis^12^. This probably explains some of the remaining false positives in our model.

In the independent ALD validation cohort (*n* = 63), the F2 and I2 proteomics models yielded higher ROC–AUC values than any of the ten in-class comparators available in the cohort, with an ROC–AUC of 0.87 and 0.81, respectively (Fig. 5b,c and Supplementary Tables 20 and 21). For steatosis only, CAP outperformed our S1 model with an ROC–AUC of 0.88 compared against 0.79.

To further assess proteomics model performance we calculated net reclassification improvement (NRI)^49^, a metric widely used to quantify how well a new model reclassifies subjects compared with a baseline model, using the test set in both the discovery and validation cohorts (Methods). This resulted in net advantages of the F2, I2 and S1 proteomics models compared with all their comparators at their clinically recommended cutoffs (NRI > 0; Supplementary Table 22). Specifically, the F2 proteomics model performed better in ruling out disease than TE and the ELF blood test but was less accurate at ruling in disease, resulting in a net advantage of this model. Similarly, SWE and the APRI index ruled out significant fibrosis slightly better than the F2 model but were weaker at ruling in significant fibrosis. Our F2 proteomics model performed better at both ruling in and ruling out disease than the FIB-4 index. Likewise, our I2 proteomics panel was substantially better than the AST/ALT ratio in ruling in hepatic inflammatory activity but not for ruling out, still resulting in an overall higher performance than AAR. The S1 proteomics panel was slightly better than CAP in ruling in any steatosis, while the two techniques were comparable for ruling out steatosis (Supplementary Table 22).

### Proteomics models are accurate prognostic measures

**Extended Data Fig. 7:**
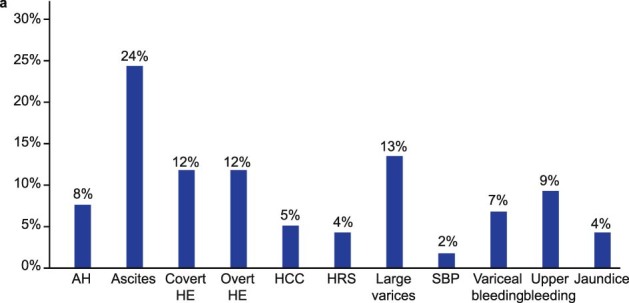
Distribution of individual components of the liver-related event composite endpoint. Percentage of occurrence of each liver-related events during the follow-up period. Abbreviations: AH, alcoholic hepatitis; HCC, hepatocellular carcinoma; HE, hepatic encephalopathy; HRS, hepatorenal syndrome; SBP, spontaneous bacterial peritonitis. Source data

Using nationwide Danish electronic health records, we extracted longitudinal, clinical outcome data by following 457 patients in the GALA–ALD cohort from the day of inclusion until death or end of the follow-up period (30 October 2020). We defined two primary outcomes: liver-related events (LREs, a composite endpoint comprising 11 types of outcome (Supplementary Table 23)) and all-cause mortality. During a median follow-up period of 53 months (IQR 34–74) and 2,035 person-years, 76 patients died and 85 experienced one or more LREs, the latter dominated by decompensation (89%, 106 events) caused by portal hypertension, with ascites being the commonest single manifestation (Extended Data Fig. 7). There were four cases of severe alcoholic hepatitis without concomitant evidence of decompensation related to portal hypertension (5% of all LREs), and five cases of hepatocellular carcinoma (HCC) without concomitant evidence of portal hypertension (6% of all LREs).

We compared our proteomics models against competing commercial biomarkers and histologic staging of liver fibrosis (Methods). The F2 proteomics model showed the highest discriminative accuracy for prognostication of LREs, with Harrell’s *C* = 0.900 and AUC = 0.945 (3-year) and AUC = 0.933 (5-year), significantly higher than the F3 and I2 proteomics models, with transient elastography the second highest (Fig. 5d–f and Supplementary Tables 24 and 25). For all-cause mortality, the F2 proteomics model also performed best (Harrell’s *C* = 0.789, 3- and 5-year AUC = 0.836 and 0.818, respectively) (Fig. 5g–i and Supplementary Table 24), but did not statistically outperform TE, ELF or the F3 proteomics model (Supplementary Table 26).

## Discussion

Here we set out to investigate liver and plasma proteome changes associated with liver pathophysiology, and to identify circulating proteins of diagnostic value in detection of liver fibrosis, inflammatory activity and steatosis. Specifically, we asked whether we could detect liver disease by a state-of-the-art, MS-based proteomics workflow. Indeed, the biomarker panels used for detection of the three key pathological features of liver disease identified in this study have considerably better, or at least comparable, performance compared with a comprehensive collection of existing best-in-class clinical tests as judged by F1 score, balanced accuracy and DeLong’s test on ROC–AUC. A validation cohort further reproduced the model performance of marker panels for detection of significant fibrosis and mild inflammation as determined by ROC–AUC. The NRI index further identified the specific clinical advantages of the proteomics models in comparison with existing tests. For instance, the F2 proteomics model is better at ruling out significant fibrosis than TE or the ELF blood test. Confident ruling out of disease reduces false positives, which is especially valuable in general practice where over-referral is a prominent issue. Our results indicate the feasibility of plasma proteomics for the simultaneous and accurate detection of fibrosis, inflammation and steatosis in ALD to inform clinical decision making. The combined diagnostic and prognostic information based on a single blood sample may reduce patient–hospital interactions and allow faster treatment decisions. In this study, we applied a default cutoff value of 0.5 for disease classification on patients in the validation cohort. To develop the biomarker panels into a diagnostic test in the clinic, the cutoff value can be optimized to balance sensitivity and specificity.

Thus, advances in MS-based, high-throughput proteomics hold potential for clinical translation. Inherently, this technology has the advantage of proteome-wide protein quantification making the size of the marker panels unimportant. It already shows competitive performance in detection of significant fibrosis (including F2) compared with elastography imaging modalities. To increase throughput and reduce costs, targeted proteomics assays could be developed that measure a subset of proteins in a shorter analysis time, similar to the ubiquitous and low-cost LC–MS/MS-based vitamin D tests. Another example of FDA-approved, MS-based in vitro diagnostic device is the Biotyper instrument for microbial identification, showing that MS can be deployed on a very large scale. In future, customized mass spectrometers that are easy to maintain and operate may be developed to serve as clinical diagnostic tools. The magnitude of some of the changes detected in this study is very small (for example, increases of 9% for CFH and 24% for QSOX1, and up to 82% for LGALS3BP at stage F2 compared with F0, calculated as F2/F1 – 1). For such small changes, analytical reproducibility and quantification accuracy are probably best achieved by MS-based proteomics, which is also much more specific than affinity-based methods. However, for purposes of commercialization and easy access, we do not exclude the possibility of developing immunoassay-based methods for protein marker panels or targeted combinations of immunocapture with specific and rapid MS readout.

Integration of liver and plasma proteomics resulted mainly in the identification of upregulated proteins. This observation might be due to both a larger proportion of upregulated proteins observed in the liver proteome and a higher magnitude of dysregulation in upregulated than downregulated proteins. Biologically our findings reflect the immune response and imbalanced ECM turnover, which result in dramatic liver proteome remodeling. In contrast, impaired liver synthetic function leads to less pronounced fold changes.

The marker panels underlying these models were selected in an unbiased manner, driven by their diagnostic performance. While some have been implicated in liver disease confirming previously published findings, such as C7 (ref. ^50^), LGALS3BP^51^, TGFBI^52^, ALDOB^21^, AFM^21,53^, TTR^54^, QSOX1 (ref. ^52^), F2 (ref. ^52^), APOF^55^, LBP^56^ and SERPINC1 (ref. ^57^), several have the potential to serve as new circulating biomarkers, including ASAP1, CLEC3B, CFH, ITIH4, ATRN, PEPD and SERPINF1. The high prognostic performance of the F2 and I2 proteomics models suggests that the underlying proteins may be related to observed LREs. Panel proteins of these two models have molecular functions linked to the immune system (C7, CFH, FCN2, ORM1), ECM organization, cell–cell adhesion and migration (TGFBI, LGALS3BP, QSOX1), coagulation cascade (F2, SERPINC1, PROC), transport in the bloodstream (TTR, ORM1 and APOF), metabolism (ALDOB) and HCC (prognostic markers CLEC3B, ASAP1). Our data suggest that pathophysiological characteristics traditionally associated with compensated and decompensated cirrhosis could occur at earlier stages of ALD and drive disease progression—for example, immune dysfunction (characterized by both increased systemic inflammation and immunodeficiency), coagulation disequilibrium (resulting in microthrombi in the liver sinusoids), protein calorie malnutrition and dysbalanced ECM turnover, where collagen formation supersedes collagen degradation^58^. The lower prognostic performance of the S1 proteomics model is consistent with observational studies showing that biopsy-confirmed liver fibrosis is the main prognostic predictor of LREs and overall mortality^23,59,60^ while simple steatosis carries little prognostic information.

The strengths of this study include a large, deeply phenotyped cohort with liver biopsy as reference standard for diagnostic markers and complete, multi-year outcome events derived from nationwide electronic health records. Both the derivation and validation ALD cohorts consisted of consecutively recruited patients, representing the full spectrum of patients with asymptomatic chronic ALD before the onset of decompensation. We thereby avoid the spectrum and selection bias seen in many biomarker discovery studies^61^. The controls we used for evaluation of the specificity of model performance were not taking any medication, had no chronic disease and had not received antibiotics for infections within the previous 6 months at least. In this healthy cohort our proteomics panels accurately predicted the absence of liver disease, an encouraging result for potential screening purposes. The F2 and I2 proteomics panels also accurately ruled out disease in the subgroup of patients with ALD and low liver stiffness. Our proteomics models confidently ruled out fibrosis and inflammation in the at-risk but liver-healthy subset, and also correctly diagnosed participants in the independent validation cohort.

Among the limitations to this study, first there is currently no universally accepted system for scoring the entire clinical spectrum of ALD. The international consensus to date has been to apply NAFLD grading and staging systems to ALD because of shared histological presentations^12,23^. We chose NAS because it is the most commonly used scoring system for NAFLD and ALD. However, some prognostic features of ALD, such as cholestasis and Mallory–Denk bodies, are not considered in NAFLD grading^62^. These features are more common in hospitalized patients with alcoholic hepatitis, where inflammation and hepatocyte cell death are more severe than those observed in our cohort^62^. Post hoc we evaluated a subgroup of 106 participants for cholestasis, and found that none had ductular cholestasis and only 9% showed evidence of mild canalicular cholestasis. We cannot exclude that specific proteomics markers of cholestasis may have been omitted but, given the scarcity of these specific histological features, it is unlikely to change the overall conclusions of our study. Second, we cannot exclude the possibility of some blood contamination when performing pairwise correlations between liver and plasma, because liver perfusion is applicable only in mouse models. If this occurred, the true correlation between plasma and tissue proteome changes could be less than that observed. Accordingly, some of the dysregulated proteins in plasma may not be liver specific but could have stemmed from other affected organs, such as the gut or adipose tissue, but this would not have affected their diagnostic value. Third, we have not yet demonstrated the cost effectiveness of our approach, because it is not intended to be a final product ready for implemention in clinical practice.

Our results also have implications for the development of potential ALD treatment options. For example, 20% of upregulated tissue proteins were associated with signal transduction pathways such as the receptor tyrosine kinase, and some less-studied, but also druggable, signaling pathways in ALD, including G-protein-coupled receptors. Furthermore, codysregulated proteins in the liver and plasma represent diverse aspects of chronic liver disease such as immune and inflammatory responses, cell adhesion, ECM organization and protease inhibition. Some of these molecules may likewise be targets for pharmaceutical development. Even more detailed functional comparisons of plasma and tissue changes could be enabled by in-depth liver atlases^63^. In summary, this study confirms previous findings and provides potential protein targets that are of diagnostic, prognostic and therapeutic value in ALD. The marker panels could be applicable to other liver disease etiologies such as NAFLD, with which they share common histological presentations. However, this generalization would require studies in cohorts of comparable size, disease spectrum and clinical characterization.

## Methods

### Ethical approval

The study protocol was approved by the ethics committee for the Region of Southern Denmark (nos. S-20160006G, S-20120071, S-20160021 and S-20170087) and is registered with both the Danish Data Protection Agency (nos. 13/8204, 16/3492 and 18/22692) and Odense Patient Data Exploratory Network (under study identification nos. OP_040 and OP_239 (open.rsyd.dk/OpenProjects/da/openProjectList.jsp)). The study protocol (still ongoing) for the ALD validation cohort was registered at clinicaltrials.gov (ID: NCT03308916). The study was conducted according to the principles of the Declaration of Helsinki, and oral and written informed consent was obtained from all participants.

### Participant recruitment and clinical data collection

For the GALA–ALD cohort (*n* = 459) we recruited patients consecutively through standard referrals to three outpatient liver clinics, from two municipal alcohol rehabilitation centers and through a community call to screen for ALD in the Region of Southern Denmark. Inclusion criteria in the GALA–ALD cohort were a history of misuse of alcohol for >1 year (>24 g d^–1^ for women and >36 g d^–1^ for men), age 18–75 years and informed consent to undergo a liver biopsy. We excluded patients with evidence of decompensated cirrhosis (obvious ascites, known esophageal varices, previous decompensation), concurrent liver disease other than alcohol related, severe alcoholic hepatitis, debilitating disease with an expected survival of <1 year, hepatic congestion or cholestasis evidenced by ultrasound, or inability to comply with the study protocol (Supplementary Table 27).

We performed percutaneous liver biopsy with a 17G Menghini suction needle (Hepafix, Braun) on patients in the GALA–ALD cohort, except for a subset (*n* = 97) because we revised the protocol in 2016 to remove biopsy in those whose liver stiffness measurements with transient elastography indicated no or minimal fibrosis (FibroScan <6.0 kPa). We staged liver fibrosis according to the Kleiner score^25^: F0, no fibrosis; F1, portal or periportal fibrosis only; F2, perisinusoidal fibrosis in combination with portal or periportal fibrosis; F3, bridging fibrosis; and F4, cirrhosis. We also assessed liver histology according to NAS–CRN^25^, which is widely adopted and the gold standard scoring system for ALD. The resultant histologic staging and scoring serve as the basis for disease-stage-dependent bioinformatics analysis and machine learning-based modeling: fibrosis on a scale of 0–4 (F0–4), inflammatory activity score on a scale of 0–5 (I0–5, summed lobular inflammation and hepatocyte ballooning) and steatosis on a scale of 0–3 (S0–3). Among those who had undergone liver biopsy (*n* = 361), 56% had significant fibrosis (≥F2) and 26% had advanced fibrosis (≥F3), creating a cohort that well represented the early stages of the disease where new diagnostic tools are needed.

We chose the NAFLD activity score because it is the most commonly used scoring system for NAFLD and ALD, and there is currently no universally accepted scoring system for the entire histological spectrum of ALD. Very recently, the study of alcohol-related liver disease in Europe (SALVE consortium) published a proposal for a histological scoring system specifically developed for ALD^62^. The major difference between SALVE and the NAS score is the addition of scoring for canalicular and ductular cholestasis, while lobular inflammation and ballooning are included in both scoring systems for inflammatory activity. We checked the subset of 106 biopsies in the GALA–ALD cohort, which were evaluated for the new SALVE features, canalicular and ductular cholestasis. Canalicular cholestasis was described in ten patients (9%) while ductular cholestasis was not found in any of the biopsies.

For the GALA–HP cohort (*n* = 137) we recruited healthy controls partly matched for age (40–75 years), gender and BMI, through online advertisements and social media in the Region of Southern Denmark. We excluded healthy controls in case of any medication or any chronic disease among other exclusion criteria (Supplementary Table 27).

The ALD validation cohort (*n* = 63) consisted of independent participants in a population screening study, initiated after the conclusion of the GALA–ALD study. Inclusion criteria for this study were a history of misuse of alcohol for >5 years (≥24 g d^–1^ for women and ≥36 g d^–1^ for men) and age 30–75 years. We excluded patients with evidence of decompensated liver disease with clear signs of cirrhosis: obvious ascites, overt hepatic encephalopathy and large esophageal varices with/without variceal bleeding, among other exclusion criteria (Supplementary Table 27). A slightly higher proportion of patients in this cohort had significant fibrosis (≥F2, 70% as compared with 56% in the discovery cohort), but these contributed mainly to mild stages (F2 and F3) and a lower proportion of cirrhosis (F4). In contrast, there were slightly fewer patients with mild hepatic inflammation (≥I2) (47% as compared with 54% in the discovery cohort and a median inflammatory activity score of 1 compared with 2).

### Patient characteristics

Characteristics of patients with GALA–ALD (*n* = 459): median age 57 years (IQR 13), 76% male, BMI 27.4 kg m^–2^ (IQR 6.7), model for end-stage liver disease (MELD) score 6 (IQR 2, range 6–18), liver stiffness measured by FibroScan 6.5 kPa (IQR 6.8, range 1.5–75) and liver fibrosis stage from biopsies: F0/1/2/3/4, 36/124/106/27/67. Not all biopsies contained sufficient tissue for adequate assessment of steatosis, ballooning and lobular inflammation, and therefore the following includes 352 patients only: liver steatosis score from biopsy: S0/1/2/3, 156/85/72/39; liver ballooning score from biopsy: 0/1/2, 178/108/66; liver lobular inflammation score from biopsy: 0/1/2/3, 80/160/84/28. In total, 94 patients had severe fibrosis or cirrhosis (F3, *n* = 27, 8%; F4, *n* = 67, 19%). Of these, median MELD score was 8 (IQR 7–10), median Child–Pugh score was 5 (IQR 5–6) and, when classified into A/B/C, the corresponding proportions were 72/20/2 and 77/21/2%. The two patients with Child–Pugh C both had elevated bilirubin, low albumin and mild ascites as evidenced by ultrasound. We did not perform a biopsy but included these patients in the project because they did not have known liver disease before inclusion.

Characteristics of GALA–HP participants (*n* = 137): median age 53 years (IQR 13), 63% male, BMI 26.1 kg m^–2^ (IQR 4.7), no medication, no chronic diseases, no recent antibiotics (at least 6 months), MELD score 6 (IQR 1, range 6–10), liver stiffness measured by FibroScan 4.3 kPa (IQR 1.7, range 2.6–9.7) kPa. We did not conduct a liver biopsy in the healthy control cohort, for ethical reasons.

Characteristics of ALD validation cohort patients (*n* = 63): median age 58 years (IQR 14), 86% male, BMI 30.2 kg m^–2^ (IQR 8.1), MELD score 7 (IQR 2, range 2–13); liver stiffness measured by FibroScan 9.2 kPa (IQR 5.4, range 5–58); liver fibrosis stage from biopsy: F0/1/2/3/4, 4/15/21/13/9; liver ballooning score: 0/1/2, 37/16/6; liver lobular inflammation score: 0/1/2/3, 10/30/18/1; liver steatosis score: S0/1/2/3, 19/20/12/8. In total, 22 patients had severe fibrosis or cirrhosis (F3, *n* = 13, 21%; F4, *n* = 9, 15%); of these, median MELD score was 7 (IQR 6–8) and the Child–Pugh score was 5 (classified as A) in all patients (except for three, in which Child–Pugh score calculation was not available).

### Plasma proteome sample preparation

Plasma samples were prepared using a modified protocol based on a previously published plasma proteome-profiling pipeline on an automated liquid handling system (Agilent Bravo) in a 96-well plate format^22^. Specifically, 45 µl of lysis buffer (10 mM tris(2-carboxyethyl)phosphine, 40 mM chloroacetamide, 100 mM Tris, pH 8.5) was added to 5 µl of blood plasma sample to achieve a tenfold dilution (plasma plate). The diluted solution was thoroughly mixed by pipetting 50 times up and down, for a volume of 40 µl. The plate was then centrifuged up to 300*g*, and 5 µl of the tenfold-diluted plasma was pipetted into a new 96-well plate filled with 15 µl of lysis buffer in each well (digestion plate). The digestion plate was heated to 95 °C for 10 min, followed by cooling to room temperature for 5 min. The denatured protein mixture was digested at 37 °C for 2 h after the addition of 20 µl of a freshly prepared 0.05 µg µl^–1^ trypsin/LysC mixture into each well, to a final volume of 40 µl (1 µg of enzyme to 40 µg protein, with an estimated 80 µg µl^–1^ protein concentration in plasma). Enzymatic digestion was quenched by the addition of 64 µl of 0.2% trifluoroacetic acid (TFA) then thoroughly mixed by pipetting 20 times up and down. A total of 500 ng of digestion mixture was loaded onto a disposable Evotip C18 trap column (Evosep Biosystems) according to the manufacturer’s instructions. Briefly, Evotips were wetted with 2-propanol, activated with 0.1% formic acid in acetonitrile, equilibrated with 0.1% formic acid and then loaded using centrifugal force at 1,000*g* for 2 min. Evotips were then washed with 0.1% formic acid followed by the addition of 200 µl of 0.1% formic acid to prevent drying.

### Liver proteome sample preparation

Snap-frozen liver biopsies were cryopulverized in a Covaris cryoPREP Dry Pulverizer and collected in a glass tube. Approximately 1 mg (in some cases <1 mg) of tissue powder was transferred to an Eppendorf tube, with the addition of 150 μl of SDC reduction and alkylation buffer (PreOmics). The homogenate was then heated at 95 °C for 10 min, vortexed at 1,200 r.p.m. on a thermo mixer (Eppendorf) to denature proteins and subsequently sonicated using a water bath sonicator (Diagenode Bioruptor) at full power for 30 cycles at 30-s intervals, followed by a second round of sonication using the Covaris Adaptive Focused Acoustics sonication system (Covaris). Protein content was determined by tryptophan assay, and a volume containing 50 μg of protein was digested overnight with trypsin and LysC (1 µg of enzyme to 50 µg of protein) at 37 °C, 1,200 r.p.m. on a thermo mixer. The digestion mixture was acidified to a final concentration of 0.1% TFA to quench the digestion reaction. Peptide concentration was estimated using Nanodrop, and 20 μg of peptide mixture was purified by solid phase extraction in a Stage-Tip format (SDB–RPS material, two 14G plugs), washed with isopropanol/1% TFA and 0.2% TFA (200 μl each, centrifuged at 1,500*g* with a three-dimensionally printed centrifugal block). Peptides were eluted with 60 μl of 80% acetonitrile/1% ammonia and dried at 60 °C using a SpeedVac centrifuge (Eppendorf, Concentrator plus). Dried peptides were dissolved and sonicated in 5% acetonitrile/0.1% TFA. Peptide concentration was measured using Nanodrop, and 500 ng of purified peptides was injected for LC–MS/MS analysis.

### LC–MS/MS analysis

The acquisition of samples was randomized to avoid bias. In single-shot plasma proteome analysis, the peptide mixture was partially eluted from Evotips with <35% acetonitrile and analyzed with an Evosep One LC system (Evosep Biosystems^27^) coupled online to an Orbitrap Exploris 480 mass spectrometer (Thermo Fisher Scientific). Eluted peptides were separated on a 8-cm-long PepSep column (150 µm inner diameter packed with 1.5 μm of Reprosil-Pur C18 beads (Dr Maisch)) in a standard preset gradient method (21 min, 60 samples per day) and electrosprayed with a stainless emitter (30 µm inner diameter) at 2.2 kV. Data were acquired in DIA mode (Supplementary Table 28). Each acquisition cycle consisted of a survey scan at a resolution of 60,000 (normalized automatic gain control target (AGC) of 300% and 100 ms injection time (IT), tune v.3.1.279.9), and 33 DIA cycles with dynamic isolation windows at a resolution of 30,000 at 200 *m/z* (with AGC target set to ‘standard’ and maximum injection time mode set to ‘auto’). Higher-energy collisional dissociation (HCD) fragmentation was set to a normalized collision energy of 30%. In all scans, PhiSDM^29^ was enabled with 100 iterations and spectral type was set to centroid.

In single-shot liver proteome analysis, purified peptides were measured using LC–MS instrumentation consisting of an EASY-nLC 1200 system (Thermo Fisher Scientific) interfaced online with a Q Exactive HF-X Orbitrap (Thermo Fisher Scientific). Peptides were separated on 42.5-cm high-performance LC columns (75 µm inner diameter packed with 1.9 µm of ReproSil-Pur C18-AQ beads (Dr Maisch)). For each LC–MS/MS analysis, around 0.5 µg of peptides was injected for 100-min gradients. Peptides were loaded in buffer A (0.1% formic acid) and eluted with a linear 82-min gradient of 3–23% of buffer B (0.1% formic acid and 80% (v/v) acetonitrile), followed by an 8-min increase to 40% of buffer B. Gradients were then increased to 98% of buffer B within 6 min, which was maintained for 4 min. Flow rates were kept at 350 nl min^–1^. Re-equilibration was performed for 4 μl of 0.1% buffer A at a pressure of 980 bar. Column temperature was maintained at 60 °C using an integrated column oven (PRSO-V2, Sonation). Data were acquired using an optimized DIA method enabled by MaxQuant.Live (v.1.0)^28^ in which the scan protocol was defined. Each acquisition cycle consisted of a survey scan at a resolution of 60,000 with an AGC of 3 × 10^6^ and IT of 100 ms, followed by 66 DIA cycles (Supplementary Table 28) at a resolution of 15,000 with an AGC of 3 × 10^6^ and IT of 22 ms in the range 300–1.650 *m/z*. HCD fragmentation was set to normalized collision energy of 27%. In all scans, PhiSDM^29^ was enabled with 100 iterations and spectral type was set to ‘centroid’.

### MS data analysis

Data-independent acquisition spectra in the liver biopsy dataset were analyzed with Spectronaut v.13, and the plasma dataset was analyzed with Spectronaut v.15.4 (ref. ^64^). Default settings were used unless otherwise noted. Data filtering was set to ‘Qvalue’. ‘Cross run normalization’ was enabled with the strategy of ‘local normalization’ based on rows with ‘Qvalue complete’. FDR was set to 1% at both the protein and peptide precursor levels. A previously generated deep fractionated plasma data-dependent acquisition (DDA) library and liver DDA library were used in the targeted analysis of DIA data for plasma and liver datasets against the human reference proteome database (2018 release, 21,007 canonical and 72,792 additional sequences).

### Preprocessing of liver and plasma proteomics datasets

Proteome datasets of liver and plasma were filtered for 60% valid values across all samples (proteins with >40% missing values were excluded from downstream statistical analysis), with the remaining missing values imputed by drawing random samples from a normal distribution with downshifted mean by 1.8 s.d. and scaled s.d. (0.3) relative to that of abundance distribution of all proteins in one sample. Specifically, in total 536 proteins were quantified in the plasma proteomes and filtering for 60% valid values across all samples resulted in a dataset of 311 proteins with a data completeness of 96%. Assessed on 13 quality control samples, median workflow coefficient of variation was 19% across a 2-week measurement period (Extended Data Fig. 2). Protein intensities were then log_2_ transformed for downstream statistical and bioinformatics analysis. The liver proteome was preprocessed in the same way, and further details are provided above (Extended Data Fig. 2).

### Differential abundance analysis

Differentially abundant proteins were determined by ANCOVA controlling for the common covariates age, BMI, sex and abstinence status at inclusion. We also controlled for the effects of steatosis when assessing the effect of fibrosis and inflammation on the liver and plasma proteomes, and vice versa. Histological stages include a five-grade fibrosis score (F0–4, denoted as Kleiner), a six-grade inflammation score (NAS inflammation, I0–5) combining lobular inflammation and ballooning, and a four-grade steatosis score (NAS steatosis, S0–3). A python script (v.3.7) based on the open-source statistical package pingouin.ancova (pingouin v.0.4.0) was developed to handle ANCOVA in proteomics data and to control for multiple hypothesis testing. A protein was considered significantly differentially abundant across a given condition if the ANCOVA-derived, FDR-adjusted *P* value by Benjamini–Hochberg was <0.05. Numbers of samples in this analysis can be found in Supplementary Table 29, and significant proteins and ANCOVA statistics are provided in Supplementary Tables 1 and 7. Differential abundance across disease stage is presented as a heatmap generated by Perseus computational software (v.1.6.5.0)^65^.

### Proteome correlation to histology scores

Spearman partial correlation, controlling for the same covariates as ANCOVA, was performed to assess protein–histology score correlation. Correlation was considered significant if the FDR-adjusted *P* value by Benjamini–Hochberg was <0.05 and the absolute value of correlation coefficient *r* ≥ 0.3. A python script based on the open-source statistical package pingouin.partial_corr was developed to handle partial correlation in proteomics data and to control for multiple hypothesis testing. The number of samples in this analysis can be found in Supplementary Table 29 while significant proteins, corresponding *P* values and Spearman correlation coefficients are provided in Supplementary Tables 4–6 and 9–11. Protein abundance in regard to dependence on disease stage is presented as bar plots generated in the Jupyter Notebook environment.

### Pairwise liver–plasma proteome correlation

Pairwise correlation was performed to assess the correlation between paired liver biopsy and plasma across the patient cohort. Significance level was controlled at an FDR-adjusted *P* value by Benjamini–Hochberg of <0.05 and an absolute value of correlation coefficient *r* > 0.3. A python script based on the open-source statistical package pingouin.pairwise_corr was developed to handle pairwise correlation in proteomics data and to control for multiple hypothesis testing. Significant proteins and corresponding *P* values, Pearson correlation coefficients and annotations from the Human Protein Atlas are provided in Supplementary Table 12, while the number of samples in this analysis can be found in Supplementary Table 29. Selected significantly correlating proteins are presented as scatter plots, with MS signal in liver biopsy as a function of that in plasma.

### Functional annotation and enrichment analysis

Ontology enrichment analysis in the liver proteomics dataset was performed with ClueGo, a plug-in in Cytoscape (v.3.6.1), with default settings. A customized reference set containing 4,651 unique genes (quantified in this study) was used in Fisher’s exact test. Term significance was corrected by Benjamini–Hochberg with FDR <1%. Both Gene Ontology term fusion and grouping were activated. Significantly enriched Reactome and associated proteins are provided in Supplementary Tables 2 and 3.

Liver‐specific proteins were annotated according to the Human Protein Atlas, which defines ‘liver-enriched’, ‘group-enriched’ and ‘liver-enhanced’ proteins with at least fivefold higher messenger RNA levels in liver compared with all other tissues, at least fivefold higher mRNA levels in a group of between two and seven tissues compared with the remainder and at least fivefold higher mRNA levels in the liver compared with average levels in all tissues, respectively.

### Machine learning models

The machine learning part of this manuscript was conducted with the intention to identify biomarker panels for identification of different types of hepatic lesions. A graphic representation of the overall machine learning workflow can be found in Supplementary Fig. 2. In brief, we first determined binary classification targets based on clinical relevance followed by feature selection for each of these classification targets. We then built logistic regression models based on the selected marker panels for each target and evaluated the model performance by both cross-validation and in the test set of a final train–test split. We benchmarked the model performance against 15 existing best-in-class clinical tests including two types of elastography, the ELF blood test, P3NP, FibroTest, FIB-4, Forns test and APRI test for fibrosis, cytokeratin-18 based markers M30 and M65 and M30/M65, together with transaminases ALT, AST and AAR for inflammation, and controlled CAP for steatosis. We then assessed model performance in regard to ruling out disease in low-incidence populations and validated it in regard to ruling in disease in an independent ALD cohort. Last, we assessed the prognostic performance of the proteomics models by performing survival analyses and prognostic analyses on patients in the GALA–ALD cohort using risk scores generated by the models.

The rationale for determining binary classification targets is as follows: we treated each pathological feature—fibrosis, inflammatory activity and steatosis—as an independent entity and aimed to identify the presence of early-stage lesions, and hence the following binary classification targets. We chose significant fibrosis (≥F2) over advanced fibrosis (≥F3) because there is an urgent clinical need to predict early-stage fibrosis and a lack of available biomarkers for this outcome. In addition, up to 20% of patients with ALD and moderate fibrosis (F2) experienced liver-related events during a median 4 years of follow-up^23^, highlighting the need to include this condition. Similarly, we chose the binary targets of inflammatory activity and steatosis based on clinical relevance: the presence of mild inflammatory activity and above (≥I2) has been shown to be a predictor of rapid progression of chronic liver disease due to alcohol abuse, but there is no evidence that clearly supports a dose–response relationship between inflammation and progression rate. The presence of any steatosis (≥S1) is a marker of the adverse effect of alcohol on the liver and constitutes a diagnostic part of alcohol-related fatty liver disease. Detection of liver steatosis at an early stage, followed by lifestyle modification and close monitoring, may benefit a person’s health on their further journey in life.

More specifically, in the GALA–ALD cohort, 360 patients had liver biopsy-verified stages of fibrosis (360), inflammation (352) and steatosis (352), as well as clinical data to varying degrees of missing values from best-in-class clinical tests. For example, SWE measurement was available for 331 individuals, M65 for 264 and CAP for 199. Two of these patients were excluded due to insufficient proteome depth having been quantified. To account for missing data across patients, and to have comparable subsets of patients when benchmarking model performance against each other, we defined a pattern of data missingness based on each patient’s pattern of availability for all considered variables, and split each train-and-test set based on this pattern of data missingness.

Proteomics data used for machine learning were processed in the same way as for statistical and bioinformatics analyses, for overall consistency. Briefly, the plasma proteome dataset was filtered for a minimum of 200 quantified proteins as the threshold of sufficient proteome depth. This was followed by filtering for 60% valid values across all samples, with the remaining missing values imputed by drawing random samples from a normal distribution with downshifted mean by 1.8 s.d. and scaled s.d. (0.3) relative to that of abundance distribution (log_2_ transformed) of all proteins within each sample. We then filtered proteins for a maximum of 30% of coefficient of variation calculated from analytical replicates (quality assessment samples) to ensure good analytical reproducibility of the selected proteins.

We determined three binary classification targets based on clinical relevance: significant fibrosis (F0–1 versus F2–4, 200 controls, 160 cases); mild inflammatory activity (I0–1 versus I2–5, 153 controls, 189 cases); and any liver steatosis (S0 versus S1–3, 156 controls, 196 cases). In addition, we included the prediction of advanced fibrosis (F0–2 versus F3–4, 266 controls, 94 cases). The proportion of positive class was 54–56% in ≥F2, ≥I2 and ≥S1 and 26% in ≥F3). We performed feature selection using a Python implementation (https://github.com/smazzanti/mrmr) of the mRMR feature selection algorithm. We assessed the relationship between model performance and number of features (from one to 50) based on fivefold, ten times cross-validation (Supplementary Fig. 3a–c). We set the maximum number of features to 50 out of practical considerations: a marker panel based on a relatively small set of proteins may be easier to translate and implement into clinical practice, and also more robust, because too many hyperparameters in a high-dimensional space may lead to an overfitting problem. We then determined the optimal number of features (or marker panels) for each classification target according to the ‘maximal F1 score using the minimal set of proteins’ principle. To show the superior performance of a composite maker panel over a single protein-based prediction model (best-performing protein), we include the comparison in Supplementary Fig. 3d. To provide an indication of feature importance in predictions, we report their coefficients in the logistic regression models in Supplementary Fig. 3e–h.

After selection of marker panels for each classification target, we evaluated their classification performance in the discovery cohort in two ways: (1) mean model performance in the test sets of fivefold ten times cross-validation (80% training and 20% test split in each of the 50 cross-validations based on the pattern of data missingness described above); and (2) model performance in the test set of a finally fitted model based on a random train–test split on which DeLong’s test was performed for the statistical comparison of AUCs. For clinical comparators we used both clinically recommended fixed cutoffs and logistic regression-determined cutoffs to ensure fair comparison. Fixed clinical cutoffs can be viewed as a threshold model that is not fitted to specific data. The metrics for comparison are precision, recall, F1 score and balanced accuracy (Supplementary Tables 15 and 17). DeLong’s test results can be found in Supplementary Table 18.

The cross-validation procedure is intended to give an overview on model performance variance from random data splits. We used the scikit-learn (sklearn library version 0.23.2) module sklearn.model_selection.RepeatedStratifiedKFold to implement the fivefold ten times cross-validation procedure. This procedure was intended to give a better representation of model performance variance from random data splits. In each fivefold cross-validation, the entire sample set in the discovery cohort was split into five smaller sets (folds 1–5), with stratification based on the pattern of data missingness described above. These five smaller sets were then partitioned into five train–test pairs by iterating each as a test set. In each of these, we trained a logistic regression model independently on the training set (four sets) and validated the resulting model on the test set (one set). This fivefold cross-validation procedure was then repeated ten times with different randomization in each repetition, resulting in a total of 50 train–test pairs (and 50 trained models), and the average model performance in the 50 test sets was then computed and presented. The ten times cross-validations were independent from each other in the sense that default parameters (except for 'solver', which we set to 'liblinear' considering the relatively small dataset) to the scikit learn linear logistic regression model were used throughout all models and no hyperparameter was optimized, and that model coefficients were determined separately in each model. The sample size in the train–test splits in the 50 cross-validation procedure is 286/72 train/test for fibrosis model F2, and 280/70 train/test for the inflammation and steatosis models. The final model is supposed to ease comparison with follow-up data on patients in the GALA–ALD cohort and to have an explicit comparison based on one model. The final model was also used to perform rule-out validation in lower-incidence populations and external validation in an independent ALD cohort (model performance is given in Supplementary Tables 19 and 20), which were not used for training or feature selection. Performance of the proteomics models in the validation cohort of ALD was likewise benchmarked against 11 best-in-class clinical tests available for the cohort with ROC–AUC as the evaluation metric (Supplementary Table 21).

Net reclassification improvement was calculated for all three proteomics models (F2, I2 and S1) against existing clinical tests at their established cutoffs based on the formula defined in the original paper^49^. Data used for NRI calculation are a combination of the test set of the finally fitted model in the derivation cohort (*n* = 72, not exposed to training) and the independent validation cohort (*n* = 63). In each comparison the F2, I2 or S1 proteomics model serves as the new model while the clinical test in comparison serves as the baseline model. The formula is as follows:

1 $${\mathrm{NRI}} = P\left( {{\mathrm{up}}|{\mathrm{event}}} \right) - P\left( {{\mathrm{down}}|{\mathrm{event}}} \right) + P\left( {{\mathrm{down}}|{\mathrm{nonevent}}} \right) - P\left( {{\mathrm{up}}|{\mathrm{nonevent}}} \right)$$

2 $${\mathrm{NRI}}_{\mathrm{e}} = P\left( {{\mathrm{up}}|{\mathrm{event}}} \right) - P\left( {{\mathrm{down}}|{\mathrm{event}}} \right)$$

3 $${\mathrm{NRI}}_{{\mathrm{ne}}} = P\left( {{\mathrm{down}}|{\mathrm{nonevent}}} \right) - P\left( {{\mathrm{up}}|{\mathrm{nonevent}}} \right)$$

For assessment of prognostic capability, we extracted liver-related events and all-cause mortality that occurred during the follow-up period of 53 months (IQR 34–74) from 457 patients in the GALA–ALD cohort, and 2,035 person-years from electronic health records at hospitals in the Region of Southern Denmark, combined with the Danish National Registry of central personal identification numbers. We defined a liver-related event as the occurrence of any of the following: alcoholic hepatitis, varices needing treatment, variceal bleeding, ascites, spontaneous bacterial peritonitis, hepatic encephalopathy, HCC, hepatorenal syndrome, upper gastrointestinal bleeding or jaundice due to liver failure. Harrell’s *C*-index and ROC–AUC for prediction of liver-related events and all-cause mortality at 3 years, 5 years and the entire follow-up period were computed in Stata software (Stata BE v.17). We then compared the prognostic performance of the proteomics marker panels with competing commercial biomarkers and liver histological lesions as reference^23^. The number of samples used in the assessment of diagnostic and prognostic capability can be found in Supplementary Table 29.

### Reporting summary

Further information on research design is available in the Nature Research Reporting Summary linked to this article.

## Online content

Any methods, additional references, Nature Research reporting summaries, source data, extended data, supplementary information, acknowledgements, peer review information; details of author contributions and competing interests; and statements of data and code availability are available at 10.1038/s41591-022-01850-y.

## Supplementary information

Supplementary Information Supplementary Figs 1–3.

Reporting Summary

Supplementary Tables Supplementary Tables 1–29.

## Data Availability

The human reference proteome (2018 release, both canonical and additional sequences) was downloaded from the European Bioinformatics Institute database (https://ftp.ebi.ac.uk/pub/databases/reference_proteomes/). Tissue specificity annotation of proteins was downloaded from the Human Protein Atlas database (https://www.proteinatlas.org/about/download). All results from statistical and bioinformatics analysis are provided in the Supplementary Tables. Due to the need to maintain patient confidentiality, patient and proteomics data generated in this study cannot be made publicly available. Averaged protein levels in the liver and plasma proteomes, and paired protein–histologic score data, have been deposited in the GitHub repository (https://github.com/llniu/ALD-study, subfolder ALD-App), which contains the Dashboard application ALD_app.py and the datasets needed to run on a local machine. The full proteomics datasets and histologic scoring generated and/or analyzed during the current study are available from the authors upon request, to Odense Patient Data Exploratory Network (open@rsyd.dk) with reference to project ID OP_040. Permission to access and analyze data can be obtained following approval from the Danish Data Protection Agency and the ethics committee for the Region of Southern Denmark. The study protocol, standard operating procedures and patient information are also available upon request. The time frame for response to requests from the authors is within a 1-month period. When applying and processing data, certain restrictions apply: (1) a data processing agreement must be signed between the data controller and processor; (2) data must not be processed for purposes other than statistical and scientific studies; and (3) personal data must be deleted, anonymized and destroyed at the end of investigation and must not be passed on to a third party or individuals who are not authorized to access the data. Source data are provided with this paper.

## Source data

Source Data Fig. 2 Statistical source data.
Source Data Fig. 3 Statistical source data.
Source Data Fig. 4 Statistical source data.
Source Data Fig. 5 Statistical source data.
Source Data Extended Data Fig. 2 Statistical source data.
Source Data Extended Data Fig. 4 Statistical source data.
Source Data Extended Data Fig. 6 Statistical source data.
Source Data Extended Data Fig. 7 Statistical source data.

## Code availability

All python scripts used in this study can be reviewed and downloaded at the Github repository https://github.com/llniu/ALD-study. Specifically, the subfolder ALD-ML contains the Jupyter Notebook ALD_ML.ipynb (scripts for the machine learning pipeline), ALD_ML_STATA.ipynb (scripts for survival analysis in STATA) and ALD_META_ML.ipynb (scripts for comparison of state-of-the-art machine learning classifiers); the subfolder ALD-PA contains the Jupyter Notebook ALD_PA.ipynb (scripts for proteomics data processing, ANCOVA, partial correlation and pairwise correlation analysis); and the subfolder ALD-App contains the Dashboard application ALD_app.py and the datasets needed to run on a local machine.
