# Supplementary material for: Noninvasive proteomic biomarkers for alcohol-related liver disease
Source: Nat Med. 2022 Jun 2;28(6):1277–87. doi: 10.1038/s41591-022-01850-y (PMC9205783; doi:10.1038/s41591-022-01850-y)
Supplement: Supplementary file 1 — Supplementary Figs 1–3. [file 41591_2022_1850_MOESM1_ESM.pdf]

nature portfolio

https://doi.org/10.1038/s41591-022-01850-y

Supplementary information

Noninvasive proteomic biomarkers for alcohol-related liver disease

In the format provided by the authors and unedited

Supplementary Figure 1

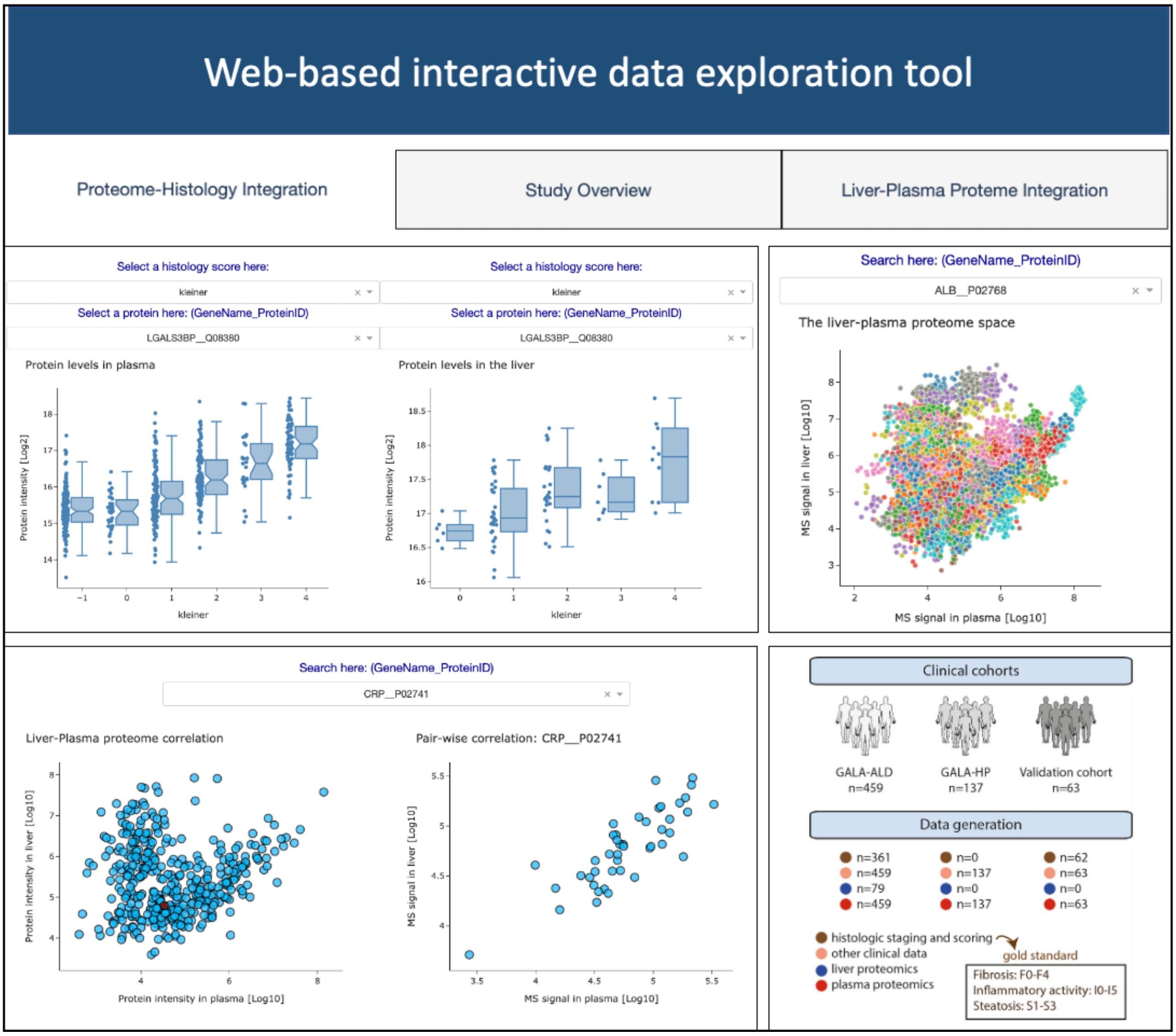

Supplementary Figure 1. An interactive, web-based data exploration tool built with the open-source Dash framework. This tool enables dynamic data visualization and query for results, including protein abundance as a function of disease severity (liver histology scores), projection of plasma proteome to the liver space, and pairwise correlation between liver and plasma samples.

Supplementary Figure 2

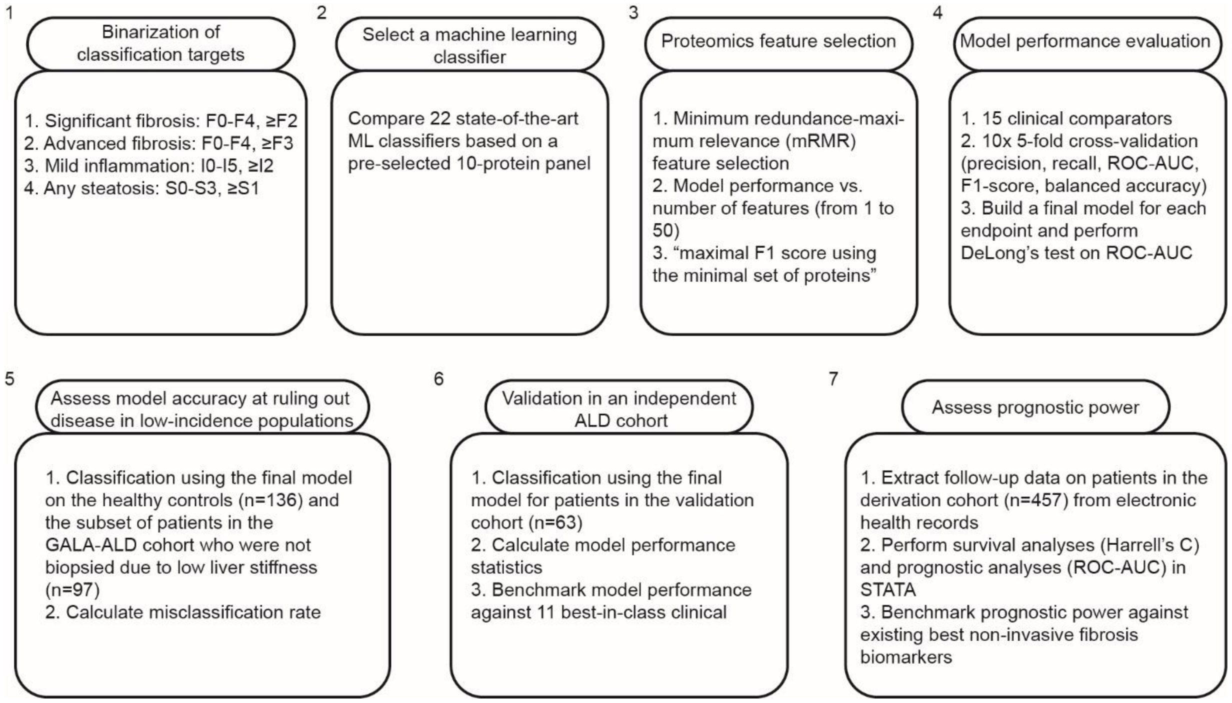

Supplementary Figure 2. Overview of the machine learning pipeline. This pipeline includes strategies for the determination of classification targets, selection of the machine learning classifier, feature selection and the evaluation of proteomics models' diagnostic and prognostic capabilities.

Supplementary Figure 3

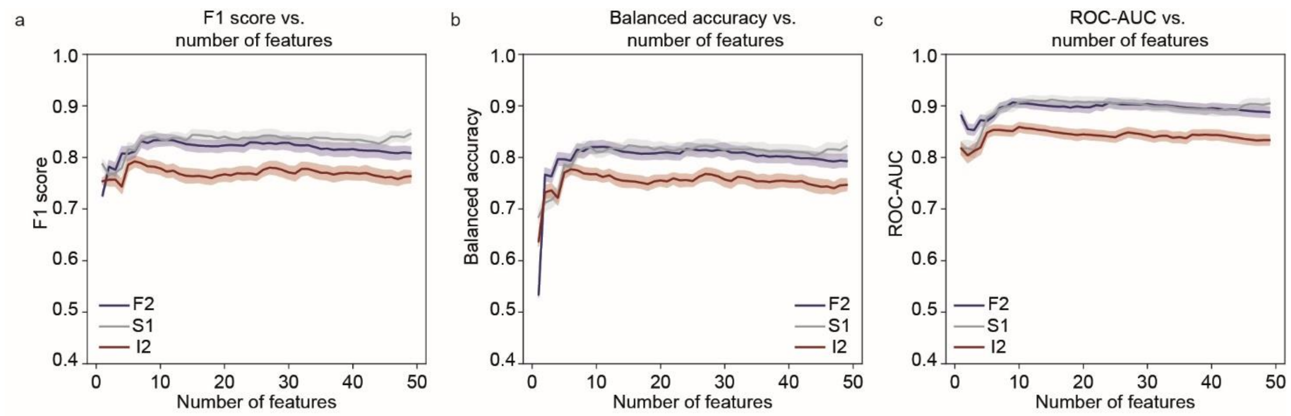

d

Classfication target	Nr. features	Precision	Recall	F1 score	Balanced accuracy	ROC-AUC	Nr. observations in total
F2	9	0.86	0.82	0.83	0.82	0.91	358
F2	1	0.57	0.99	0.73	0.53	0.88	358
I2	6	0.80	0.79	0.79	0.78	0.85	350
I2	1	0.62	0.96	0.75	0.64	0.82	350
S1	12	0.84	0.84	0.84	0.82	0.91	350
S1	1	0.68	0.94	0.79	0.68	0.82	350

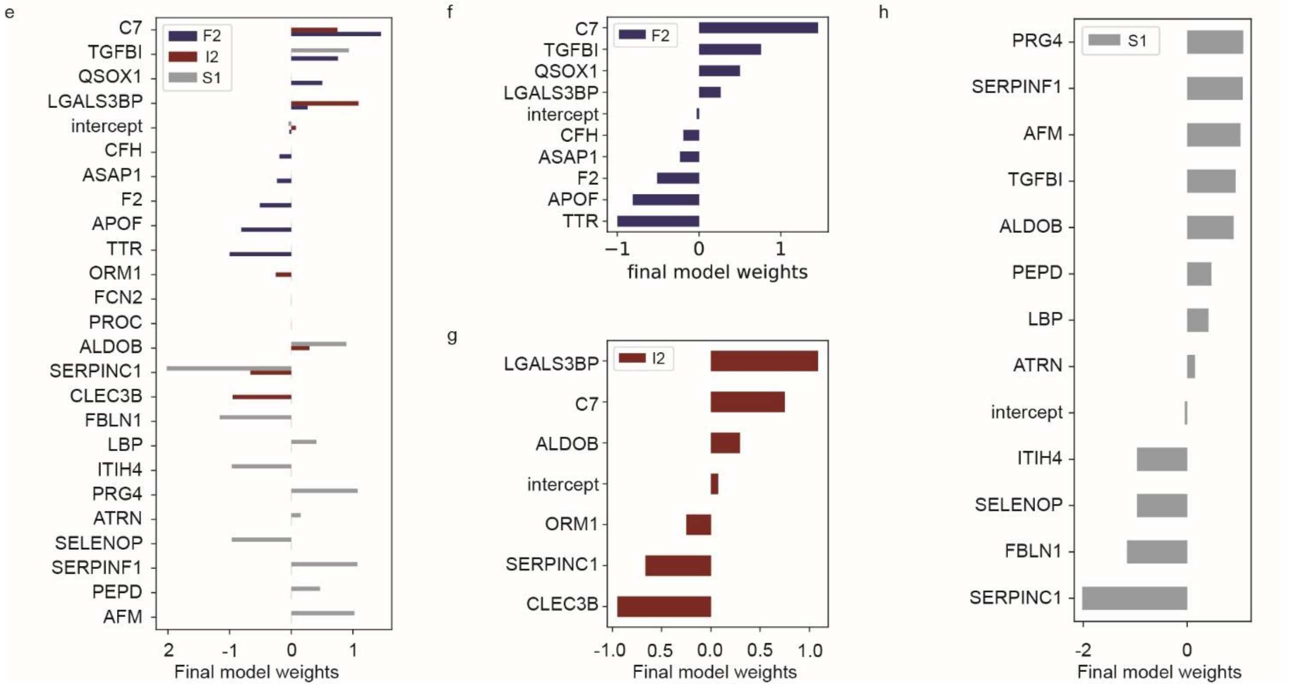

Supplementary Figure 3. Number of features in the marker panels and feature weights in logistic regression models. a-c. Value of F1 score (a), balanced accuracy (b) and ROC-AUC (c) for increasing number of features from 1 to 50. Solid line stands for the mean from 5-fold ten times cross validation, with the upper and lower border showing 95% confidence intervals. **d.** Comparison of classification performance between single-protein (best performing) and panel-protein. **e.** Feature weights of all proteins forming marker panels for the three prediction endpoints (F2, I2 and S1). **f-h.** Feature weights of proteins in the marker panel for predicting significant fibrosis (f), mild inflammation (g), and any steatosis (h).
