# Supplementary material for: Noninvasive proteomic biomarkers for alcohol-related liver disease
Source: Nat Med. 2022 Jun 2;28(6):1277–87. doi: 10.1038/s41591-022-01850-y (PMC9205783; doi:10.1038/s41591-022-01850-y)
Supplement: Supplementary file 2 — Reporting Summary [file 41591_2022_1850_MOESM2_ESM.pdf]

nature research

Corresponding author(s): Matthias Mann

Last updated by author(s): April 22, 2022

Reporting Summary

Nature Research wishes to improve the reproducibility of the work that we publish. This form provides structure for consistency and transparency in reporting. For further information on Nature Research policies, see our Editorial Policies and the Editorial Policy Checklist.

Statistics

For all statistical analyses, confirm that the following items are present in the figure legend, table legend, main text, or Methods section.

n/a	Confirmed	
☐	☒	The exact sample size (*n*) for each experimental group/condition, given as a discrete number and unit of measurement
☐	☒	A statement on whether measurements were taken from distinct samples or whether the same sample was measured repeatedly
☐	☒	The statistical test(s) used AND whether they are one- or two-sided<br>*Only common tests should be described solely by name; describe more complex techniques in the Methods section.*
☐	☒	A description of all covariates tested
☐	☒	A description of any assumptions or corrections, such as tests of normality and adjustment for multiple comparisons
☐	☒	A full description of the statistical parameters including central tendency (e.g. means) or other basic estimates (e.g. regression coefficient) AND variation (e.g. standard deviation) or associated estimates of uncertainty (e.g. confidence intervals)
☒	☐	For null hypothesis testing, the test statistic (e.g. *F*, *t*, *r*) with confidence intervals, effect sizes, degrees of freedom and *P* value noted<br>*Give P values as exact values whenever suitable.*
☒	☐	For Bayesian analysis, information on the choice of priors and Markov chain Monte Carlo settings
☒	☐	For hierarchical and complex designs, identification of the appropriate level for tests and full reporting of outcomes
☒	☐	Estimates of effect sizes (e.g. Cohen's *d*, Pearson's *r*), indicating how they were calculated

Our web collection on statistics for biologists contains articles on many of the points above.

Software and code

Policy information about availability of computer code

Data collection	MaxQuant.Live (version 1.0) was used to create the mass spectrometry acquisition methods. The commercial software Spectronaut (version 13 for liver proteomics data and version 15.4 for plasma proteomics data) was used to perform protein identification and quantification.
Data analysis	The open source Perseus computational platform (version 1.6.5.0) was used to perform hierarchical clustering. Cytoscape (version 3.6.1, open source) and the built-in ClueGo app was used for the functional annotation and enrichment analysis. The commercial software Stata BE version 17) was used for the survival- and prognostic analyses. Remaining statistical analysis and the machine learning pipeline were performed using custom scripts built on packages (version controlled) within the Python ecosystem. Specifically, the pingouin pacakge (version 0.4.0) was used for ANCOVA, partial correlation and pair-wise correlation; and the scikit learn library (version 0.23.2) was used for the machine learning analysis. All Python scripts can be reviewed and downloaded at the GitHub repository https://github.com/llniu/ALD-study. Specifically, the sub-folder ALD-ML contains the Jupyter Notebook ALD_ML.ipynb (scripts for the machine learning pipeline), ALD_ML_STATA.ipynb (scripts for the survival analysis in STATA) and ALD_META_ML.ipynb (scripts for comparing state-of-the-art machine learning classifiers); and the sub-folder ALD-PA contains the Jupyter Notebook ALD_PA.ipynb (scripts for proteomic data processing, ANCOVA, partial correlation and pair-wise correlation analysis).

For manuscripts utilizing custom algorithms or software that are central to the research but not yet described in published literature, software must be made available to editors and reviewers. We strongly encourage code deposition in a community repository (e.g. GitHub). See the Nature Research guidelines for submitting code & software for further information.

Data

Policy information about availability of data

All manuscripts must include a data availability statement. This statement should provide the following information, where applicable:

- Accession codes, unique identifiers, or web links for publicly available datasets
- A list of figures that have associated raw data
- A description of any restrictions on data availability

The human reference proteome (2018 release, both canonical and additional sequences) was downloaded from the European Bioinformatics Institute database (https://ftp.ebi.ac.uk/pub/databases/reference_proteomes/). Tissue specificity annotation of proteins was downloaded from the Human Protein Atlas database (https://www.proteinatlas.org/about/download). All results from statistical and bioinformatics analysis were provided in the Supplementary Tables. Due to the need to maintain patient confidentiality, the patient and proteomics data generated in this study cannot be made publicly available. Averaged protein levels in the liver and plasma proteome, and paired protein-histologic score data have been deposited in the GitHub repository https://github.com/llniu/ALD-study sub-folder ALD-App, which contains the Dashboard application ALD_app.py and the datasets needed to run on a local machine. The full proteomics datasets and histologic scoring generated during and/or analysed during the current study are available from the authors upon request to Odense Patient Data Exploratory Network (open@rsyd.dk) with reference to project ID OP_040. Permission to access and analyze data can be obtained following approval from Danish Data Protection Agency and the ethics committee for the Region of Southern Denmark. The study protocol, standard operating procedures and patient information are also available upon request. The time frame for response to requests from the authors is within a one-month period. When applying and processing the data, certain restrictions apply including (i) a data processing agreement must be signed between the data controller and processor; (ii) the data must not be processed for purposes other than statistical and scientific studies; (iii) personal data must be deleted, anonymized, destroyed at the end of investigation and must not be passed on to third-party or individuals who are not authorized to access the data.

Field-specific reporting

Please select the one below that is the best fit for your research. If you are not sure, read the appropriate sections before making your selection.

☒ Life sciences ☐ Behavioural & social sciences ☐ Ecological, evolutionary & environmental sciences

For a reference copy of the document with all sections, see nature.com/documents/nr-reporting-summary-flat.pdf

Life sciences study design

All studies must disclose on these points even when the disclosure is negative.

Sample size	When designing the study protocol (prior to recruiting participants), we planned to work with multiomics datasets using systems biology. Consequently, the sample size was based on experience from prior -omics studies in humans. To detect small effect sizes under parametric assumptions at 0.05 significance level and 0.75 statistical power, we estimate that 364 samples will be required for comparing proteomics profiles using statistical power analysis principles outlined by Cohen (Statistical power analysis for the behavioral sciences, 2nd edition, 1988). To adjust for non-parametric tests, the corresponding minimum required samples size using Pitman's Asymptotic Relative Efficiency is 381 participants. To provide us with enough statistical power to detect small effect sizes even when testing a handful of outcome variables and controlling the false discovery rate at 0.05, while striking a balance between exclusions/drop-outs and not including an unnecessary amount of participants, we planned to enroll more than 400 alcohol over-users and less than 500. However, we would like to emphasize that traditional ways of calculating statistical power do not strictly apply to omics research, where strong patterns have been identified using small sample sizes.
Data exclusions	In total four participants were excluded from the downstream analysis due to insufficient proteome depth (total number of quantified proteins should be > 200). This criterion was set after evaluating the overall proteomics dataset quality.
Replication	The experiments for both the discovery cohort and validation cohort were performed independently once. The identified protein marker panels and the derived logistic regression models for predicting liver fibrosis, inflammation and steatosis in the discovery cohort was validated in the independent cohort with n=63.
Randomization	Random assignment of participants into experimental groups is not relevant to this study, as it is a cross-sectional study that aims to identify proteins that correspond to liver pathology. In the downstream statistical analysis, patients are grouped based on liver histologic grading and scoring . Acquisition of the proteomics data was randomized to avoid systematic bias during the measurement.
Blinding	The pathologist who scored the liver biopsy samples were blinded to clinical patient parameters other than age and gender. The investigators who performed proteomics sample preparation and data acquisition were not blinded to clinical patient parameters as randomization in proteomics data generation was anyway performed to avoid systematic bias during the measurement and blinding is thus not relevant.

Reporting for specific materials, systems and methods

We require information from authors about some types of materials, experimental systems and methods used in many studies. Here, indicate whether each material, system or method listed is relevant to your study. If you are not sure if a list item applies to your research, read the appropriate section before selecting a response.

Materials & experimental systems

n/a	Involved in the study
☒	☐ Antibodies
☒	☐ Eukaryotic cell lines
☒	☐ Palaeontology and archaeology
☒	☐ Animals and other organisms
☐	☒ Human research participants
☐	☒ Clinical data
☒	☐ Dual use research of concern

Methods

n/a	Involved in the study
☒	☐ ChIP-seq
☒	☐ Flow cytometry
☒	☐ MRI-based neuroimaging

Human research participants

Policy information about studies involving human research participants

Population characteristics

GALA-ALD patient characteristics (n=459): Median age 57 (IQR 13) years, 76% male, BMI 27.4 (IQR 6.7) kg/m2, MELD-score 6 (IQR 2, range 6-18), liver stiffness measured by FibroScan 6.5 (IQR 6.8, range 1.5-75) kPa. Liver fibrosis stage from biopsies: F0/1/2/3/4 = 36/124/106/27/67. Not all biopsies contained enough tissue for adequate assessment of steatosis, ballooning and lobular inflammation, the following does therefore encompass 352 patients. Liver steatosis score from biopsy: S0/1/2/3 = 156/85/72/39. Liver ballooning score from biopsies: 0/1/2 = 178/108/66. Liver lobular inflammation score from biopsies: 0/1/2/3 = 80/160/84/28. 94 patients have severe fibrosis or cirrhosis (F3 n=27, 29%; F4 n=67, 71%). Of these, median MELD score is 8, IQR 7-10. Median Child-Pugh score is 5, IQR 5-6, and when classified into A/B/C the proportions are: 72/20/2 = 77%/21%/2%. The two patients with Child C both had elevated bilirubin, low albumin and mild ascites evidenced by ultrasound. We abstained from a biopsy but included them in the project because they did not have known liver disease prior to inclusion.

GALA-HP participant characteristics (n=137): Median age 53 (IQR 13) years, 63% male, BMI 26.1 (IQR 4.7) kg/m2, no medication, no chronic diseases, no recent antibiotics (at least 6 months), MELD-score 6 (IQR 1, range 6-10), liver stiffness 4.3 (IQR 1.7, range 2.6-9.7) kPa. We did not conduct a liver biopsy in the healthy control cohort for ethical reasons.

ALD validation cohort patient characteristics (n=63): Median age 58 (IQR 14) years, 86% male, BMI 30.2 (IQR 8.1) kg/m2, MELD-score 7 (IQR 2, range 2-13), liver stiffness measured by FibroScan 9.2 (IQR 5.4, range 5-58) kPa. Liver fibrosis stage from biopsy: F0/1/2/3/4 = 4/15/21/13/9. Liver ballooning score: 0/1/2 = 37/16/6. Liver lobular inflammation score: 10/30/18/1. Liver steatosis score: S0/1/2/3 = 19/20/12/8. 22 patients have severe fibrosis or cirrhosis (F3 n=13, 59%; F4 n=9, 41%). Of these, the median MELD score is 7 IQR 6-8, and the Child-Pugh score is 5 (classified as A) in all patients (except for three, in which Child-Pugh score calculation was not available).

Recruitment

For the GALA-ALD cohort (n=459), we recruited patients consecutively through standard referrals to three outpatient liver clinics, from two municipal alcohol rehabilitation centers, and through a community call to screen for alcohol-related liver disease in the Region of Southern Denmark. Inclusion criteria in the GALA-ALD cohort are a history of excessive use of alcohol for more than 1 year (>24 g per day for women and >36 g per day for men), age 18–75 years, and informed consent to undergo a liver biopsy. We excluded patients with evidence of decompensated cirrhosis (obvious ascites, known esophageal varices, prior decompensation), concurrent liver disease other than alcohol-related, severe alcoholic hepatitis, debilitating disease with an expected survival of less than one year, hepatic congestion or cholestasis evidenced by ultrasound, or inability to comply with the study protocol.

For the GALA-HP cohort (n=137), we recruited healthy controls, partly matched for age, gender and BMI through online adverts and social media in the Region of Southern Denmark (age 40-75 years). We excluded healthy controls in case of any medication or any chronic disease.

The ALD validation cohort (n=63) consists of independent participants in a population screening study, initiated after the conclusion of the GALA-ALD study. Inclusion criteria for this study are a history of harmful use of alcohol for more than 5 years (≥24 g per day for women and ≥36 g per day for men), age 30-75 years. We excluded patients with evidence of decompensated liver disease with clear signs of cirrhosis: obvious ascites, overt hepatic encephalopathy, large esophageal varices with/without variceal bleeding.

In terms of potential bias, half of the patients in the GALA-ALD cohort were recruited from secondary care and the other half from primary care. A disadvantage with this is that the cohort is not specific or representative to neither clinical setting. However, the advantage is that the cohort covers the full spectrum of ALD. We recruited patients consecutively, specifically to avoid selection bias. Another potential bias is that we did not perform percutaneous liver biopsy on a subset of patients (n=98) in the GALA-ALD cohort because we revised the protocol in 2016 to abstain from biopsy in those whose liver stiffness measurements with transient elastography indicated no or minimal fibrosis (FibroScan below 6.0 kPa). The potential consequence of this is that we may have excluded patients that were classified as false negatives by transient elastography. Nonetheless, for differential abundance analysis in both plasma and liver tissue, we used only the patients with biopsy-verified disease stages, making it unlikely to impact our results.

Ethics oversight

The study protocol was approved by ethics committee for the Region of Southern Denmark (ethical IDs: S-20160006G, S-20120071, S-20160021, S-20170087) and registered with the Danish Data Protection Agency (13/8204, 16/3492, 18/22692).

Note that full information on the approval of the study protocol must also be provided in the manuscript.

Clinical data

Policy information about clinical studies

All manuscripts should comply with the ICMJE guidelines for publication of clinical research and a completed CONSORT checklist must be included with all submissions.

Clinical trial registration	The GALA-ALD and GALA-HP cohorts were registered with Odense Patient Data Exploratory Network under study identification number OP_040 and OP_239 (open.rsyd.dk/OpenProjects/da/openProjectList.jsp). The study protocol for the ALD validation cohort was registered at clinicaltrials.gov ID NCT03308916.
Study protocol	Study protocol can be obtained by contact to open@rsyd.dk
Data collection	GALA-ALD: Single center data collection, at Center for Liver Research, Odense University Hospital, Denmark. Inclusion and recruitment period: April 2013 to September 2018.<br>GALA-HP: Single center data collection, at Center for Liver Research, Odense University Hospital, Denmark. Inclusion and recruitment period: May 2016 to March 2018<br>ALD-Validation cohort: Single center data collection, at Center for Liver Research, Odense University Hospital, Denmark.
Outcomes	Primary outcome of this study includes proteins whose abundance levels in the plasma correlate with stages of liver fibrosis. Correlation was assessed by Spearman correlation adjusted for multiple hypothesis testing. Fibrosis staging was performed according to the Kleiner scoring system. Secondly we correlated the plasma proteome with hepatic steatosis and inflammatory activity (sum of ballooning and lobular inflammation) using Spearman correlation adjusted for multiple hypothesis testing. Scoring of hepatic steatosis and inflammatory activity were performed according to the NAFLD activity score. Further secondary outcomes were: (a) ability of the proteomics marker panels to rule out in the GALA-HP cohort by calculating the correct negative assignments by the logistic regression models that were built based on the identified biomarker panels. (b) the diagnostic performance of the proteomics marker panels compared to liver stiffness measured by transient elastography, enhanced liver fibrosis test, and indirect markers of fibrosis such as FIB-4, APRI, Forns, by comparing the F1 score, balanced accuracy and area under the receiver-operating characteristics curve (AUROC) of these tests/model in detecting significant fibrosis, mild inflammatory activity and any steatosis. (c) Investigate the prognostic power of our proteomic marker panels assessed by Harrell's C index and AUROC in predicting liver-related events and all-cause mortality, and compared them with existing non-invasive markers and the liver histological lesions as reference. (d) to validate the performance of discovery cohort-derived protein marker panels in an independent validation cohort by assessing AUROC of the proteomics models for the validation cohort, and comparing the performance with in-class existing clinical tests.
